# Furin as target for suppression of mosquito-borne viruses

**DOI:** 10.1186/s12985-026-03127-z

**Published:** 2026-03-11

**Authors:** Alejandra Centurión, Bodunrin Omokungbe, Markus Oberpaul, Ludwig Dersch, Sabrina Stiehler, Cross Chambers, Marcus Lechner, Tim Lüddecke, Andreas Vilcinskas, Torsten Steinmetzer, Kornelia Hardes

**Affiliations:** 1 LOEWE Centre for Translational Biodiversity Genomics (LOEWE-TBG), Frankfurt am Main, Germany; 2 Department of Pest and vector insect control, Fraunhofer Institute for Molecular Biology and Applied Ecology IME, Branch of Bioresources, Giessen, Germany; 3 Department of Biodiversity, Fraunhofer Institute for Molecular Biology and Applied Ecology IME, Branch for Bioresources, Giessen, Germany; 4 Institute for Insect Biotechnology, Justus-Liebig University, Giessen, Germany; 5 BMBF Junior Research Group in Infection Research ASCRIBE, Giessen, Germany; 6 Center for Synthetic Microbiology (SYNMIKRO), University of Marburg, Marburg, Germany; 7 Institute of Pharmacology, University of Marburg, Marburg, Germany; 8 Institute of Pharmaceutical Chemistry, University of Marburg, Marburg, Germany

**Keywords:** Mosquitoes, Aedes albopictus, Host factor, Proprotein convertase, Arbovirus, Semliki Forest virus, Antivirals, Inhibitor, Cell culture

## Abstract

**Background:**

Mosquitoes are the main vectors of arboviruses, which infect millions of people every year. These viruses depend on host factors, such as the proprotein convertase furin, for replication. While the interactions between arboviruses and furin have been widely studied in mammals, little is known about furin homologs and their role in virus replication in mosquitoes.

**Methods:**

We performed a comparative analysis of the sequences and predicted structures of human and other dipteran furin with their mosquito homologs. We used RT-qPCR to determine the mRNA expression of the identified furin genes. We synthesized the FITC-labeled furin inhibitor MI-1190 to analyze the uptake in C6/36 cells, larvae, and female mosquitoes. Then, we tested the toxicity of peptidomimetic furin inhibitors (MI-1148, MI-1554, and MI-1851) in vitro through cellular ATP quantification and in vivo by adding the inhibitor to the breeding water of larvae and microinjection of females. Finally, we evaluated their antiviral efficiency by quantifying the relative fluorescence generated by the viral reporter expression in cell culture and female mosquitoes.

**Results:**

We identified two furin encoding genes (FLP1 and two FLP2 transcripts) and confirmed their mRNA expression in all developmental stages of *Aedes albopictus* and two of its cell lines. The inhibitor MI-1190 was successfully taken up in C6/36 cells, as well as by early larval stages and adult female mosquitoes. The three selected inhibitors significantly curtailed the spread of Semliki Forest virus in cell culture, thereby demonstrating their antiviral efficacy in mosquito cells. However, the antiviral effect observed in vitro did not translate in vivo, where the effect of furin inhibitor MI-1851 showed only a minor impact.

**Conclusions:**

Identifying and characterizing host factors from mosquitoes as antiviral targets is a complementary step towards developing new strategies to combat arbovirus transmission and address the ongoing global health challenge.

**Supplementary Information:**

The online version contains supplementary material available at 10.1186/s12985-026-03127-z.

## Background

Vector-borne diseases are a significant global threat to public health, placing 80% of the population at risk [1]. Mosquito-borne viruses such as Dengue virus (DENV), Zika virus (ZIKV), West Nile virus (WNV), and Chikungunya virus (CHIKV) are of particular concern due to their continuous expansion and the limited availability of specific treatments and vaccines [2–5]. The primary method to prevent mosquito-borne epidemics relies on insect vector control [6, 7]. However, the use of chemical pesticides is often associated with negative effects on non-target insects [8] and encourages the emergence of resistant mosquito populations [9, 10].

Understanding the molecular interactions between viruses and their mosquito hosts is crucial to developing new strategies to block arboviral transmission. A key step in the replication of many viruses is the proteolytic activation of viral envelope glycoproteins by host proteases, which facilitates membrane fusion and cell entry [11, 12]. One of such host proteases is the proprotein convertase furin, that has a key role in the activation of various precursors of growth factors, hormones, matrix metalloproteinases, plasma proteins, and receptors [12, 13]. This ubiquitous type-I transmembrane protein contains a Ca^2+^-dependent subtilisin-like serine protease domain and preferentially cleaves its substrates after the multibasic motif R-X-(R/K)-R↓-X [13]. Prominent cellular substrates of furin in humans include the precursors of insulin-like growth factor, albumin, and transforming growth factor β1 [14]. Several arboviruses exploit furin to cleave and activate their surface proteins as prerequisite for their replication [12].

While the role of furin in viral replication is well documented in mammals, relatively little is known about furin homologs in insects, particularly mosquitoes. Studies carried out in lepidopteran cells and larvae have shown that endogenous insect furin was biologically active and correctly processed a hemagglutinin precursor of H7N1 introduced using a baculovirus vector [15, 16]. In the Diptera order, including flies and mosquitoes, two homologs of human furin, Dfur1 and Dfur2, have been identified in the fruit fly *Drosophila melanogaster* [17]. These homologs have shown to correctly process numerous substrates of human proprotein convertases [17, 18]. In the mosquito *Aedes aegypti*, several furin-like enzymes have been predicted [19], with two of them being homologous to *Drosophila* furin [20]. One enzyme was found to be nearly identical to a previously described vitellogenin convertase, responsible for yolk protein formation, crucial for vitellogenesis [21]. Furthermore, the furin homolog of Dfur1 in *Aedes albopictus* C6/36 cells efficiently cleaved the precursor protein PE2 of Sindbis virus and generated mutants containing arginine, serine, phenylalanine, histidine, asparagine, or aspartic acid in P1’ corresponding to + 1 position, confirming a similar cleavage pattern [20]. However, the expression profiles, substrates, and biological relevance of mosquito furin remain poorly understood.

Given the essential role of furin in the viral replication cycle, synthetic furin inhibitors potentially represent a promising strategy to prevent and treat arbovirus infections. We have previously described various peptidomimetic substrate analog furin inhibitors [22–30]. Among them, the tight-binding inhibitor MI-1148 (4-Guanidinomethyl-phenylacetyl-arginine-*tert*-leucine-arginine-4-amidinobenzylamide) [28], that inhibited the replication of numerous furin-dependent viruses in cell culture, including highly pathogenic avian influenza viruses (H5N1 and H7N1), CHIKV, WNV, DENV-2 (Dengue virus serotype 2), canine distemper virus, and Semliki Forest virus (SFV) [28, 29, 31]. Although it is a very effective inhibitor in vitro, it showed significant toxicity in mice [27]. This prompted the synthesis of further furin inhibitors with an improved toxicity profile, including MI-1554 [27] and MI-1851 [25]. Both demonstrated improved safety profiles while maintaining strong antiviral potency.

In this study, we aimed to examine whether targeting furin in mosquitoes would have comparable antiviral effects to those reported in mammalian cells. To test this, we first identified *Ae. albopictus* furin homologs of human and other dipteran furins, and determined the corresponding mRNA profiles in its life stages and the aedine cells C6/36 and U4.4 via RT-qPCR. We synthesized the FITC-tagged furin inhibitor MI-1190 to study the uptake and localization of the inhibitor in the C6/36 cell line, as well as early larval stages and female mosquitoes. Then, we evaluated the toxicity of the three furin inhibitors MI-1148, MI-1554, and MI-1851 in vitro and in vivo. Lastly, we assessed the antiviral efficacy of the compounds in aedine cells and females by analyzing the spread of a reporter-expressing SFV in their presence. Our results reveal the potential of furin inhibitors as a tool to reduce the replication of furin-dependent arboviruses in mosquitoes.

## Materials and methods

### Identification of furin proteins, phylogenetic tree, and sequencing alignments

Putative furin homologs in Diptera were screened in the UniProt reference proteomes set 2023_05. All Diptera proteins annotated with a “Peptidases S8 Protein convertases Kexins Furin-like” domain (cd04059, Conserved Domains Database [32]) were considered. *Culex pipiens* was not part of the UniProt reference set. The amino acid sequences were therefore taken from NCBI RefSeq assembly GCF_016801865.2 (*Culex pipiens* pallens). *Homo sapiens* furin (UniProt ID P09958) was added as outgroup for the phylogenetic analysis. Sequences were aligned using muscle v5.2 and a phylogenetic tree reconstructed using IQ-TREE v2.0.7 [33] with 1000 bootstraps and automated ModelFinder to determine the best-fit model using *H. sapiens* furin as outgroup. Based on the Corrected Akaike Information Criterion and the Bayesian Information Criterion, the VT substitution model with empirical base frequencies, a proportion of invariable sites and a discrete Gamma model with 4 rate categories [34] (VT + F+I+G4) was identified as best model and therefore used for maximum likelihood calculations. The phylogenetic tree was visualized using ITOL v7 [35]. Note that protein A0A7R8V1S3 (*Hermetia illucens*) appears at an isolated long branch. It is likely not relevant for this study but nevertheless shown for the sake of completeness as it contains a cd04059 domain. Its absence in the alignment does not influence the reconstructed phylogenetic tree or clade support to any significant extent (data not shown). UniProt IDs from *Ae. albopictus* were manually mapped to RefSeq assembly GCF_035046485.1 (A0A182HA94 to XP_062713265.1/FLP1 and A0A182GZ77 to XP_062713309.1/FLP2X1 and XP_062713310.1/FLP2X2) to extract the respective protein-coding sequences (for primer design). Notably, the RefSeq assembly of *Ae. albopictus* and *C. pipiens* depicts two isoforms of furin-like protease 2 (XP_039440028.1/A0A182GZ77), which we term X1 and X2 here. In both cases, isoform X2 differs by a deletion of three amino acids in the cd04059 domain (VSR at position 279 and 236, respectively).

Gene sequences of furin like proteases from *Ae. albopictus*, *D. melanogaster*, and *Ae. aegypti* sourced from NCBI (Table S1) were compared by aligning sequences. The sequence alignments were carried out using Geneious v.10.2.6 with global alignment with free end gaps and a 65% cost matrix.

### Expression of furin in aedine cells and *Ae. albopictus* developmental stages

For RNA extraction, we used 200 first-instar larvae, 50 second-instar larvae, 30 third- and fourth-instar larvae, 25 pupae, 10 female mosquitoes, 10 blood-fed female mosquitoes, and 10 male mosquitoes, 25 midguts of blood-fed and non-blood-fed adult females, and 3 × 10^6^ cells of C6/36 and U4.4 cells. Animal samples were homogenized using ceramic beads (2.8 mm, Qiagen) in a TissueLyser II (Qiagen) for 5 m at 30 Hz. RNA was extracted using the Monarch Total RNA Miniprep Kit (New England BioLabs) according to the manufacturer’s protocol. RNA was quantified on a NanoDrop 2000 Spectrophotometer (ThermoFisher Scientific). The A_260_/A_280_ cutoff value was set between 1.8 and 2.2. RT-qPCR was performed on a QuantStudio 3 Real-Time PCR System (Applied Biosystems, Thermo Fisher Scientific) using the Luna Universal One-Step RT-qPCR Kit (New England Biolabs) with 1 µg of extracted RNA that was reverse-transcribed and amplified. The primer sequences and amplicon size are listed in Table 1. Each reaction was heated to 55 °C for 10 m and 95 °C for 1 m followed by 40 cycles of 95 °C for 10 s and 60 °C for 1 m. Transcripts of the housekeeping gene phosphatase-2 A (PP2A, see Fig. S6 for the characterization of its expression across the different developmental stages) were used for normalization and data were analyzed via ${2}^{-\varDelta{C}_{t}}$. The common cut-off Δ*C*_*t*_ value was set to 37 and higher Δ*C*_*t*_ values were defined as “no transcripts present” [36].

**Table 1:** Sequences of the primers used in this study

Gene	NCBI accession no. transcript protein	Primer sequence	Amplicon size (bp)
Furin like protease 1 (FLP1)	XM_062857281.1 XP_062713265.1	TTCATCGAGCAGCAACCACT GCTCGTGAGGTTGAGTGTCA	184
Furin like protease 2 isoform 1 (FLP2X1)	XM_062857325.1 XP_062713309.1	CTGGTGAGTAGAAACGGTGG CGTCCGGGTCATAGTTTTGC	157
Furin like protease 2 isoform 2 (FLP2X2)	XM_062857326.1 XP_062713310.1	AAGTTCACCACAGCGCACTC GCCACCGTTCAGGTACCATT	227
Housekeeping gene protein phosphatase-2 A (PP2A)	XM_029863994.2 XP_029719854.2	TGTGTACGACCTGTTCTTGAGG CACCCGATGAAGGACAGTCT	160

### Cell culture

The *Ae. albopictus* cell lines C6/36 (kindly provided by Prof. Dr. Stefanie Becker, University of Veterinary Medicine Hannover, Germany) and U4.4 (Friedrich-Loeffler-Institute, Federal Research Institute for Animal Health, Riems, Germany) were sub-cultured at 28 °C in insect growth medium (Leibovitz L-15 Medium GlutaMAX) supplemented with 1% tryptose phosphate broth, 10% fetal calf serum, 1% MEM non-essential amino acids, and 1% penicillin/streptomycin.

Baby hamster kidney (BHK-21) cells (CLS Cell Lines Service) were maintained in Dulbecco’s modified Eagle’s medium (DMEM GlutaMAX) supplemented with 10% fetal calf serum and 1% penicillin/streptomycin at 37 °C in a 5% CO_2_ atmosphere. All media and supplements were purchased from Thermo Fisher Scientific.

### Compounds and reagents

Permethrin and ionomycin were purchased from Sigma-Aldrich (now Merck, Germany). Protected amino acids, coupling reagents, resins, further chemicals, and solvents were obtained from ORPEGEN Peptide Chemicals, Bachem Holding, Iris Biotech, PolyPeptide Laboratories, Acros, and Sigma-Aldrich.

### Mosquito rearing

We reared *Ae. albopictus* (Rimini strain, kindly provided as eggs by Dr. Hanano Yamada, Joint FAO/IAEA Center of Nuclear Techniques in Food and Agriculture, Insect Pest Control Sub-Program, Vienna, Austria) at 28 ± 3 °C and 80% ± 10% relative humidity with a 12 h photoperiod. Female mosquitoes were fed on 8% d(-)-fructose (Carl Roth) supplemented with 4-aminobenzoic acid (Merck). Defibrinated sheep blood (Thermo Fisher Scientific) was provided weekly to female mosquitoes for 1 h using the Hemotek system (Hemotek Ltd) set at 37 °C. Approximately 24 h after blood feeding, a black plastic pot with humid filter paper was placed in the rearing cage for oviposition. The filter paper with eggs was carefully dried over 10–14 d. Eggs were hatched in jars containing deoxygenated water. After hatching, ~ 500 larvae were transferred to plastic trays containing two liters of tap water. Pleco Tablets (Tetra) were provided as larval food. Pupae were collected in a jar and placed inside a rearing cage for emergence.

### Virus propagation

Infection assays were carried out using the mCherry-tagged SFV SFV6-2SG-mCherry and SFV4 (kindly provided by Prof. Dr. Andres Merits, University of Tartu, Estonia and Prof. Dr. Andreas Pichlmair, Technical University of Munich, Germany). BHK-21 cells at 90% confluency were transfected with the SFV6-2SG-mCherry plasmid in infection medium (DMEM GlutaMAX supplemented with 1% penicillin/streptomycin and 0.2% bovine serum albumin (BSA; Serva Electrophoresis)) using Lipofectamine 3000 (Thermo Fisher Scientific) according to the manufacturer’s instructions. At 48 h post-transfection, the supernatants were harvested, clarified by centrifugation at 2000 × g for 10 m at 4 °C, and stored at − 80 °C. Confluent BHK-21 cells were infected at a multiplicity of infection (MOI) of 0.001 using these virus stocks to generate the working stocks for subsequent experiments. SFV-mCherry passage information can be found in Table S3. SFV4 was propagated in BHK-21 cells using infection medium. Cells were infected at a MOI of 0.001. After 48 h, the supernatants were harvested, clarified by centrifugation at 2000 × g for 10 m at 4 °C, and stored at − 80 °C.

Viral titers were determined using a TCID_50_ assay in BHK-21 cells. Briefly, tenfold serial dilutions of each sample were inoculated onto confluent cells cultured in a 96‑well format (Greiner Bio-One) and incubated for 1 h. Then, media was removed and replenished with fresh infection media. Cells were incubated for 48 h at 37 °C in a 5% CO_2_ atmosphere before virus reporter expression and replication was quantified by fluorescence measurements and detection of cell viability using the Cytation 5 Cell Imaging Reader (Biotek). The TCID₅₀ value was calculated based on the method of Reed-Muench and was converted to estimated viral titers (PFU/mL) using the standard Poisson based approximation that 1 TCID₅₀ corresponds to 0.7 PFU.

### Synthesis of the FITC-tagged inhibitor MI-1190

Inhibitor MI-1190 was prepared by Fmoc solid-phase peptide synthesis in a 5-mL reaction vessel with a frit using Fmoc-Arg(Pbf)−2-chlorotrityl resin (0.25 g, loading 0.66 mmol/g). The Fmoc group was removed with 20% (v/v) piperdine/DMF (5 m for first cleavage and 20 m for second cleavage). For the following couplings, we used a four fold excess of the Fmoc-amino acids (Fmoc-Lys(Dde)-OH, Fmoc-Arg(Pbf)-OH, and Fmoc-4-aminomethyl-phenylacetic acid), HOBt and HBTU, respectively, and 8.0 equivalents (equiv.). DIPEA. After the final coupling of the Fmoc-4-aminomethylphenylacetic acid, subsequent Fmoc cleavage, and 4 × washing with DMF, the resin was treated with a solution of 3 equiv. *N*,*N*′-Bis-Boc-1-guanylpyrazole [37, 38] and 4 equiv. DIPEA in 2 mL DMF. The resin was shaken at room temperature overnight, followed by 3 × washing with DMF and 6 × with DCM. Subsequently, the resin was treated with 2% hydrazine/DMF (3 × 2 m) to remove the Dde-protecting group from the Lys side chain. For the coupling of Boc-β-alanine, we again used a fourfold excess of the amino acid derivative, HOBt and HBTU, respectively, and 8.0 equiv. DIPEA. The side chain protected intermediate was cleaved from the resin under mild acidic conditions (3 mL 1% (v/v) TFA/DCM, 3 × 30 m) at room temperature. The solution was immediately neutralized by adding DIPEA. The solvent was removed *in vacuo* and the oily intermediate redissolved in DMF (4 mL) and treated at 0 °C with 1 equiv. 4-amidinobenzylamine × 2 HCl, 3 equiv. 6-Cl-HOBt, 1.1 equiv. PyBOP, and 3 equiv. DIPEA. The mixture was stirred at 0 °C for 15 m and then at room temperature overnight. The solvent was removed *in vacuo* and the oily residue was treated with 2 mL cleavage cocktail (TFA/TIS/H_2_O 95:2.5:2.5 (*v*/*v*/*v*)) for 3 h at room temperature. The mixture was poured into 40 mL of cooled diethyl ether and centrifuged (Universal 16 R Hettich centrifuge, set to 4 °C at 2000 × g). The inhibitor MI-1189 (4-GMe-Phac-Arg-Lys(β-Ala)-Arg-4-Amba × 5 TFA) was washed twice with diethyl ether, and the product was purified by preparative RP-HPLC and lyophilized, yielding 71.9 mg of white powder (HPLC: 18.0 m, start at 1% solvent B (purity = 97.1%), MS ESI, positive): calc: 849.52; found *m*/*z*: 426.02 [*M* + 2 H]^2+^/2).

The inhibitor MI-1189 4-GMe-Phac-Arg-Lys(β-Ala)-Arg-4-Amba (15 mg) was dissolved in 150 µL DMF and 1 equiv. FITC and 0.5 equiv. DIPEA were added to the solution, which was stirred overnight [39]. Due to an incomplete reaction monitored by analytical HPLC, 0.75 equiv. FITC were added at the next day and the reaction was stirred for additional 5 h. The inhibitor MI-1190 (4-GMe-Phac-Arg-Lys(FITC-β-Ala)-Arg-4-Amba × 5 TFA) was purified by preparative RP-HPLC and lyophilized, yielding 14.3 mg of a yellow powder (HPLC: 28.1 m, start at 1% solvent B (purity: 93.1%), MS (ESI, positive): calc: 1238.56; found *m*/*z*: 1239.87 [*M* + H]^+)^.

### Standard analytical methods and preparative HPLC

Standard analytical reversed-phase HPLC measurements [28, 29] were carried out on a Shimadzu LC-10 A system using a Nucleodur C_18_ column (5 μm, 100 Å, 4.6 × 250 mm; Macherey-Nagel). A linear gradient with solvents A (0.1% TFA in water) and B (0.1% TFA in acetonitrile) at a flow rate of 1 mL/m was used (1% increase solvent B per m) and a detection at 220 nm. The inhibitors were purified (≥ 95% based on detection at 220 nm) using the same solvents on a preparative Varian-HPLC system equipped with a Nucleodur C_18_ column (5 μm, 100 Å, 32 × 250 mm; Macherey–Nagel) with a linear gradient of 0.5% increase of solvent B per m at a flow rate of 20 mL/m. All peptides were finally obtained as TFA salts after lyophilization. To avoid possible TFA-related side effects, we used the physiologically more acceptable HCl salts of MI-1148, MI-1554, and MI-1851. The procedure for the conversion of the salt form was described previously [25, 27]. The molecular mass of the synthesized compounds was determined on a QTrap 2000 ESI spectrometer (Applied Biosystems).

### Enzyme kinetic measurements with human furin

The *K*_i_ values of inhibitors MI-1189 and MI-1190 were determined as previously described using human furin (0.95 nm in assay) [40] and the fluorogenic substrate Phac-Arg-Val-Arg-Arg-AMC [27–29, 41] (Fig. S1).

Inhibitor MI-1189 showed a tight-binding behavior. Therefore, Eq. 1 was used to determine the apparent inhibition constant *K*_i_^*^ at the used substrate concentration, where *v*_S_ is the calculated steady state rate, *v*_0_ the velocity in absence of an inhibitor, *I*_t_ the total inhibitor concentration, and *E*_t_ the total enzyme concentration [42].

1 $$v_s = v_0 \times \frac{ \left[ \left( K_i^* + I_t - E_t \right)^2 + 4 K_i^* E_t \right]^{1/2} - \left( K_i^* + I_t - E_t \right) }{ 2\times E_t }$$

The apparent *K*_i_* value was converted into the true *K*_i_ value using Eq. 2.

2 $$K_i = \frac{K_i^*}{1 + \frac{S}{K_M}}$$

Inhibitor MI-1190 was measured under classical conditions using Eq. 3 for calculating the *K*_i_ value:

3 $$v = \frac{V_{\max}[S]}{K_M \left(1 + \frac{[I]}{K_i}\right) + [S]}$$

where *v* is the steady-state rate in the presence of a certain inhibitor concentration, V_max_ the maximal rate of the uninhibited reaction, [*S*] the substrate concentration, [*I*] the inhibitor concentration, *K*_M_ the Michaelis-Menten constant, and *K*_i_ the inhibition constant. All data were calculated with Origin v8.1.

### Uptake analysis of MI-1190 in C6/36 cells using fluorescence microscopy

In cell culture, C6/36 cells were grown on 12 mm glass coverslips in 24-well plates as described above. At a confluency of 90%, the cells were washed twice with medium and treated with the FITC-tagged furin inhibitor MI-1190 (stock 5 mM in 1:1 (v/v) H_2_O/DMSO, 10 µM final concentration) for 2 h in the incubator. The cells were then washed twice with PBS and fixed with 4% (w/v) paraformaldehyde (PFA) in PBS for 45 m. For inhibitor visualization in C6/36 cells, the coverslips were washed twice with PBS and incubated with Phalloidin-iFluor 680 Reagent (Abcam, 1X in PBS + 1% BSA per well) for 90 m in a dark chamber at room temperature.

For co-localization study using C6/36 cells, the cells were permeabilized with 0.3% Triton X-100 (Fisher Scientific) for 20 m, and blocked using blocking buffer (2% BSA, 5% glycerol, 0.2% Tween-20 in PBS) for 30 m at room temperature. The viral non-structural protein 1 was stained using rabbit serum against the protein (Prof. Dr. Andres Merits, 1:1000) and the Golgi apparatus was stained using a polyclonal goat antibody against Golgin245 (Developmental Studies Hybridoma Bank, Iowa City, IA, United States, Sinka R; J Cell Biol. 2008 Cat# Golgin245, RRID:AB_2569587, [43] 1:200) by incubating the samples for 1 h. After two washes with PBS, anti-rabbit Alexa Fluor 647– and anti-goat Alexa Fluor 555–conjugated secondary antibodies (Santa Cruz, both 1:100) were added and incubated for 1 h at room temperature. Following two additional washes with PBS, the coverslips were incubated with DAPI (4’,6-diamidino-2-phenylindole; 1 mg/mL stock solution, used at 1 µg/mL per well; Merck) for 45 m in a dark, humid chamber. Finally, the coverslips were washed five times with PBS and once with water before being mounted in Fluoroshield (Merck). The slides were dried overnight in a dark chamber. Images were acquired using a DM5000B fluorescence microscope and a S8 confocal microscope (both Leica Microsystems). Images and animations were analyzed using Leica LAS v4.13 and LASX v3.3. software.

### Uptake analysis of MI-1190 in *Ae. albopictus* larvae using fluorescence microscopy

Larvae were treated with the FITC-tagged furin inhibitor MI-1190 for 24 h (stock 5 mM in 1:1 (v/v) H_2_O/DMSO, 100 µM per well) in a 48-well plate. Larvae were examined at 1, 4, 6, 16, and 24 h post-treatment (hpt) using a Leica DM5000B fluorescence microscope. The larvae were rinsed twice with water before examination. Larvae in rearing water were used as a control group. Larvae used for time point observation were placed back in the treatment or control pool until the end of the experiment. The larvae were incubated under standard rearing conditions as described above.

### Uptake analysis of MI-1190 in *Ae. albopictus* female mosquitoes using fluorescence microscopy

Female mosquitoes were anaesthetized with CO_2_ and injected in the mesokatepisternum [44] with 101.2 nL (50.6 nL twice) of a 1 mM stock solution of MI-1190 using a Nanoject II microinjector device (Drummond Scientific) under a Stemi 508 Stereo Microscope (Carl Zeiss). The control group was injected with water. The female mosquitoes were kept in the dark under standard rearing conditions as described above. Due to the mosquitoes’ dark pigmentation, we dissected them to improve the visualization of the localization of MI-1190. The mosquitoes were passed through ethanol and then placed on glass slides with PBS for dissection of the body [45]. The fluorescence analysis was carried out at 1, 3, 6, and 24 hpt using a Leica DM5000B fluorescence microscope. Image overlays were generated using Krita v.5.2.6.

### Cytotoxicity of furin inhibitors in cell culture

The cells were seeded in 96-well plates and incubated at 28 °C until they reached 90% confluency. At this point, they were treated with the three furin inhibitors, while ionomycin, permethrin, and water were included as controls. The furin inhibitors MI-1148, MI-1554, and MI-1851 were dissolved in water (10 mM stock solutions, 100 µM per well). The FITC-tagged furin inhibitor MI-1190 was dissolved in 1:1 (v/v) H_2_O/DMSO (5 mM stock solution, 100 µM per well). Ionomycin and permethrin were dissolved in DMSO (10 mM stock solutions, 100 µM per well). The plates were incubated at 28 °C. Cell viability was assessed 48 hpt using the CellTiter-Glo Luminescent assay (Promega) according to the manufacturer’s instructions. Luminescence was recorded using black 96-well plates in a Synergy H4 microplate reader (Biotek, now Agilent). Data were normalized to the water control.

### Toxicity of the furin inhibitors in *Ae. albopictus* larvae

Larval assays were carried out in microtiter plates because only small quantities of each compound were available. For developmental larval stages L1-L2, we used 24-well plates (800 µL final volume) and for larval stages L3-L4 we used 12-well plates (1500 µL final volume). 30 larvae per developmental stage were treated with the furin inhibitors MI-1148, MI-1554, and MI-1851 (10 mM stock solution in water, 100 µM per well) and permethrin (10 mM stock solution in DMSO, 100 µM per well). Water was added to the control group. To avoid cannibalism, 1 mg of food (powdered Pleco Tablet) was added to each well. The larvae were incubated under the rearing conditions described above. Mortality data were recorded for 3 d.

### Toxicity of furin inhibitors in *Ae. albopictus* female mosquitoes

90 sugar-fed female *Ae. albopictus* mosquitoes (3–5 d old) were microinjected with 101.2 nL (50.6 nL twice) of a 1 mM stock solution of each inhibitor using the same procedure described above in the uptake analysis. Female mosquitoes in the control group were injected with water. Afterwards, the mosquitoes were maintained under the rearing conditions described above. Mortality data were recorded for 7 d.

Additionally, we tested an erythrocyte feeding method to avoid microinjection for the antiviral assay. Blood erythrocytes were obtained from centrifuged sheep blood (MiniSpin, Eppendorf, 14.1 RPM, 3 m). Plasma supernatant was removed and the pellet was resuspended in an equal volume of PBS. Females were offered erythrocytes and the inhibitor MI-1851 to a final concentration of 100 µM. Treatments containing only erythrocytes and erythrocytes with permethrin were used as controls. Females were kept under the same rearing conditions as described above. Mortality data were recorded for 5 d.

### Effect of furin inhibitors on SFV encoded mCherry expression and on SFV in cell culture

C6/36 and U4.4 cells were seeded in 96-well black µClear plates (Greiner) and grown to 90% confluency. The cells were then inoculated with SFV-mCherry at a MOI of 0.01 in non-supplemented insect growth medium (L-15 Medium GlutaMAX) for 1 h. After infection, the cells were incubated with the inhibitors (10 mM stock solution in water, 100, 10, 1, and 0.1 µM per well) in supplemented insect growth medium. Infected cells treated with water and infected cells that remained untreated were used as positive controls, whereas uninfected cells without treatment were used to measure background fluorescence. At 48 and 72 h post-infection (hpi), nuclei were stained using 8 µL of NucBlue Live ReadyProbes Reagents (Thermo Fisher Scientific) per well and incubated for 30 m for cell count analysis. After incubation, the total mean integrated red fluorescence intensity (red fluorescence) was measured using a Texas Red filter cube (1225102, Biotek, now Agilent, ex: 586/15 nm; em: 647/57 nm) and the cell count was determined by using a DAPI filter cube (1225100, Biotek, now Agilent ex: 377/50 nm; em 447/60 nm) on a Cytation 5 imaging reader (Biotek, now Agilent) via whole well image-stitching (four images at 4× magnification). The images were processed using the Gen5 Prime v.3.12 software (Biotek, now Agilent). The red fluorescence measured in the antiviral assays using SFV-mCherry was used for quantification of the virus. The total red fluorescence intensity emitted by each cell was calculated by subtracting the mean of the untreated control from each well and divided by the total cell count of the individual well.

Furthermore, C6/36 were seeded in 96-well cell culture plates (Greiner) and grown to 90% confluency. The cells were then inoculated with SFV4 at a MOI of 0.01 in non-supplemented insect growth medium (L-15 Medium GlutaMAX) for 1 h. After infection, the cells were incubated with the inhibitors (10 mM stock solution in water, 100, 10, 1, and 0.1 µM per well) in supplemented insect growth medium. At 48 hpi, the supernatants were harvested and stored at − 80 °C. Viral titers were determined using a TCID_50_ assay in BHK-21 cells as described above.

### Inhibition of E3-E2 processing by furin inhibitors in cell culture

To analyze the E3-E2 (p62) processing after MI-1148 treatment, confluent C6/36 cells and BHK-21 cells in 12-well plates were infected with SFV4 at a MOI of 1 for 1 h, followed by washing with PBS and incubation in infection medium with or without the furin inhibitor MI-1148 (25 µM) for another 24 h. Cells were washed with PBS, scratched from the bottom of the well, and lysed in 20 µL CelLytic M buffer (Merck) supplemented with aprotease inhibitor mix (G-Biosciences) according to the manufacturer's instructions. After centrifugation at 8000 × g for 3 m, the supernatant was transferred to a fresh tube. Equal amounts of protein for each sample were mixed with 6x Laemmli buffer before heating at 95 °C for 5 m. Proteins were separated by SDS-PAGE using a 4% stacking and an 8% running gel at 120 V for 90 m and transferred to a PVDF membrane (Merck) at 100 V for 1 h. The membrane was blocked for 1 h with 5% milk in Tris-buffered saline buffer supplemented with 0.1% Tween20 (TBS-T) to inhibit non-specific binding. The membrane was incubated with rabbit-anti-SFV-E2 antiserum (dilution: 1:350, obtained from Prof. Gerald McInerney, Karolinska Institutet, Stockholm, Sweden) at 4 °C overnight with shaking. Afterwards, the membrane was washed 3 times with TBS-T for 10 m with shaking and further incubated with the secondary HRP-conjugated antibody (sc-2357, Santa Cruz) for 1 h. After the membrane was washed 3 times with TBS-T, proteins were visualized using SuperSignal West Femto sensitivity substrate (ThermoFisher Scientific) using the FUSION SL image acquisition system (Peqlab).

### Effect of furin inhibitors on SFV encoded mCherry expression in vivo

We tested two different methods to infect L3-L4 larvae. Larvae were fed with scratched cells a day prior infection to avoid interference with fluorescence measurements. In a first approach, C6/36 cells were grown to confluency in 24-well plates and infected with SFV-mCherry at a MOI of 0.01. At 48 hpi, larvae were placed in the wells in groups of 10 and allowed to feed on the cells. Non-infected cells were used as control. Fluorescence signal measurements of individual larvae were made at 24 and 48 hpt. After 2 d, pooled larvae were rinsed twice and disrupted in non-supplemented L-15 media using glass beads (5 mm, Carl Roth) in a TissueLyser LT (Qiagen) for 5 m at 50 Hz, and centrifuged in a MiniSpin (14.1 RPM, 3 m). The supernatant was carefully collected from each sample. C6/36 cells were infected with the supernatants following the protocol described above. At 48 hpi, the red fluorescence was measured as described in our cell culture experiments. For the second approach, we placed 10 larvae per well in 24-well plates containing SFV-mCherry from a stock (5.20 × 10^7^ PFU/mL). Larvae on unsupplemented L-15 medium were used as controls. Larval samples were obtained and analyzed as described above in the first approach.

Females, approximately 10 d old, were fed using the Hemotek system containing 500 µL virus suspension (same concentration as used for the larval assay), 1 mL of sheep blood erythrocytes, and the inhibitor MI-1851 to a final concentration of 100 µM. The virus pH was adjusted using 1 µL of 7.5% sodium bicarbonate per 100 µL of virus stuck before being mixed with the erythrocytes [46]. The control group was fed on the same mixture but without the furin inhibitor. Engorged females were provided with either MI-1851 (100 µM) for the treatment group or water for the control group by refilling a cotton ball until the end of the experiment. Females were kept under the same rearing conditions as described above. After 3 d, mosquitoes were individually disrupted and samples were processed as described in the larval assays. The red fluorescence emitted by each cell was calculated by subtracting the mean of the untreated control from each well. Additionally, the infection rate for the treatment and virus control was calculated as the number of positive infections divided by the total number of mosquitoes tested and multiplied by 100.

### Statistical analysis

Data were analyzed using GraphPad Prism v9.1.2 for Windows (GraphPad Software). We plotted the survival curves of larvae and female mosquitoes using log-rank analysis (Kaplan-Meier method). The antiviral efficiency of the compounds in cell culture was analyzed by one-way analysis of variance (ANOVA) with Dunnett’s test between the treatments and SFV-mCherry (*p* < 0.05) and by Mann-Whitney test in the antiviral female mosquito assay.

## Results

### Furin homologs of *Ae. albopictus*

**Fig. 1:**
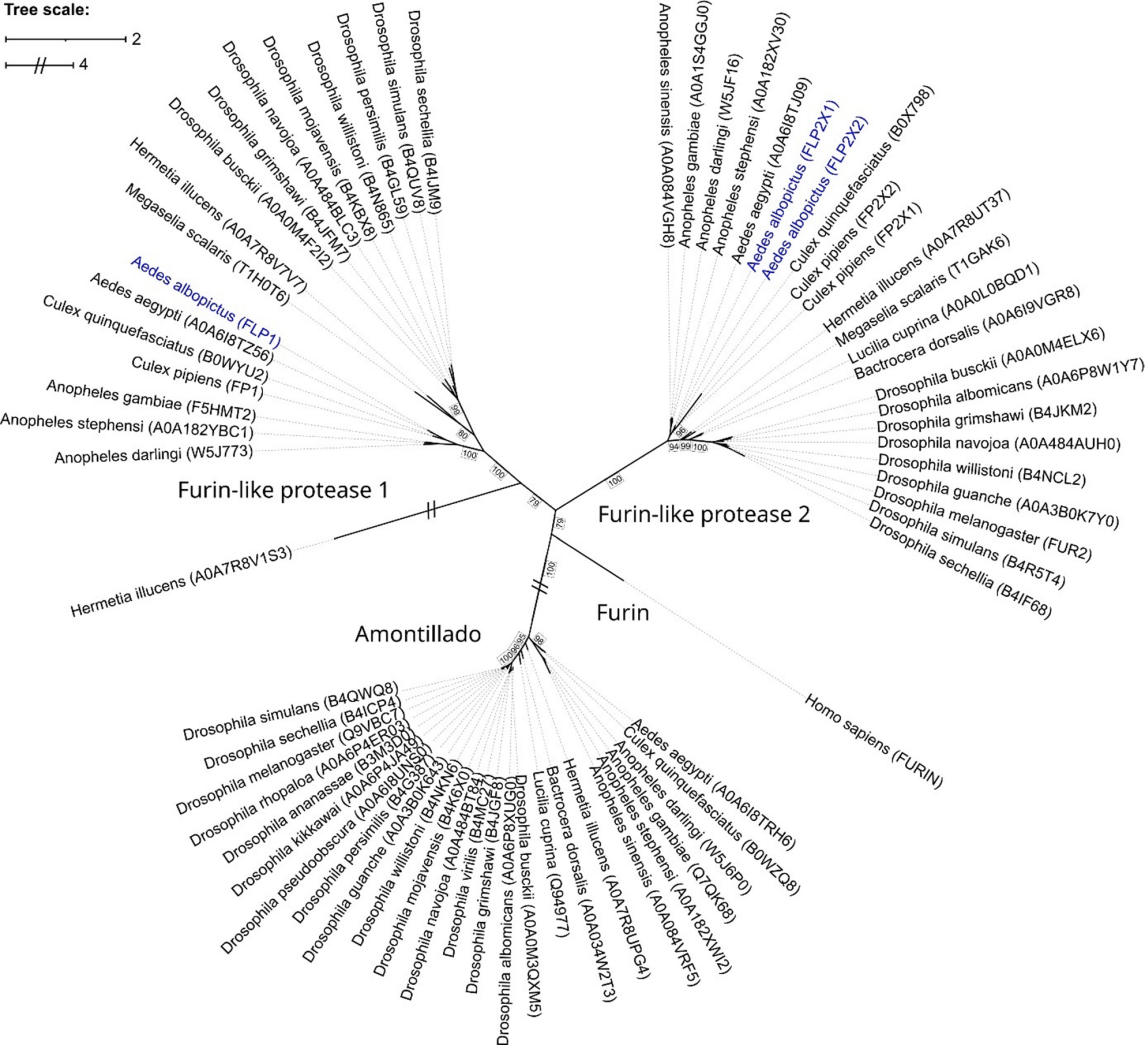
Phylogenetic relationship of proteins with peptidase S8 domains (CDD: cd04059) in Diptera based on maximum likelihood analysis. Using *Homo sapiens* furin as outgroup, three distinct clades are visible. *Ae. albopictus* was not present in the reference database (UniProt) and was thus added from another source (Vectorbase, blue). Bootstrap values were calculated over 1,000 iterations values and are shown next to the major branches. All values were above 60

To investigate the expression of furin homologs in *Ae. albopictus*, we first searched for putative furin homologs considering all Diptera proteins annotated with a “Peptidase S8 Protein convertases Kexins furin-like” domain. *Homo sapiens* furin was added to the analysis as an outgroup. We then calculated an unrooted phylogenetic tree based on maximum likelihood inference from amino acid sequences (Fig. 1), where we observed three well-supported distinct clades, the furin-like protease 1, the furin-like protease 2, and amontillado. While most proteins in the furin-like clade were annotated as “P/Homo B domain containing protein”, we found the furin-like protease 1 (FLP1) in the first one and therefore named this clade furin-like protease 1. The second clade contained two very similar proteins (isoform 1: FLP2X1, isoform 2: FLP2X2) and was therefore named furin-like protease 2. Only one representative isoform is shown in the phylogenetic tree. Additionally, we compared the nucleotide sequences from *Ae. aegypti* and *D. melanogaster* retrieved from the National Center for Biotechnology Information (NCBI) with those identified in *Ae. albopictus* (Table S1). Nucleotide sequence alignments between furin homologs FLP1 and Fur1 from *Ae. aegypti* shared 63.7% sequence identity but only 42% and 42.1% with FLP2X1 and FLP2X2, respectively. In comparison with *Drosophila* furin homologs, only Dfur1 had a notable shared sequence identity (58.3%) with FLP1, while homolog Dfur2 showed 36.5% identity to FLP1, 58.3% identity to FLP2X1 and 58.4% to FLP2X2 (Table S2).

### Expression of furin in *Ae. albopictus*

**Fig. 2:**
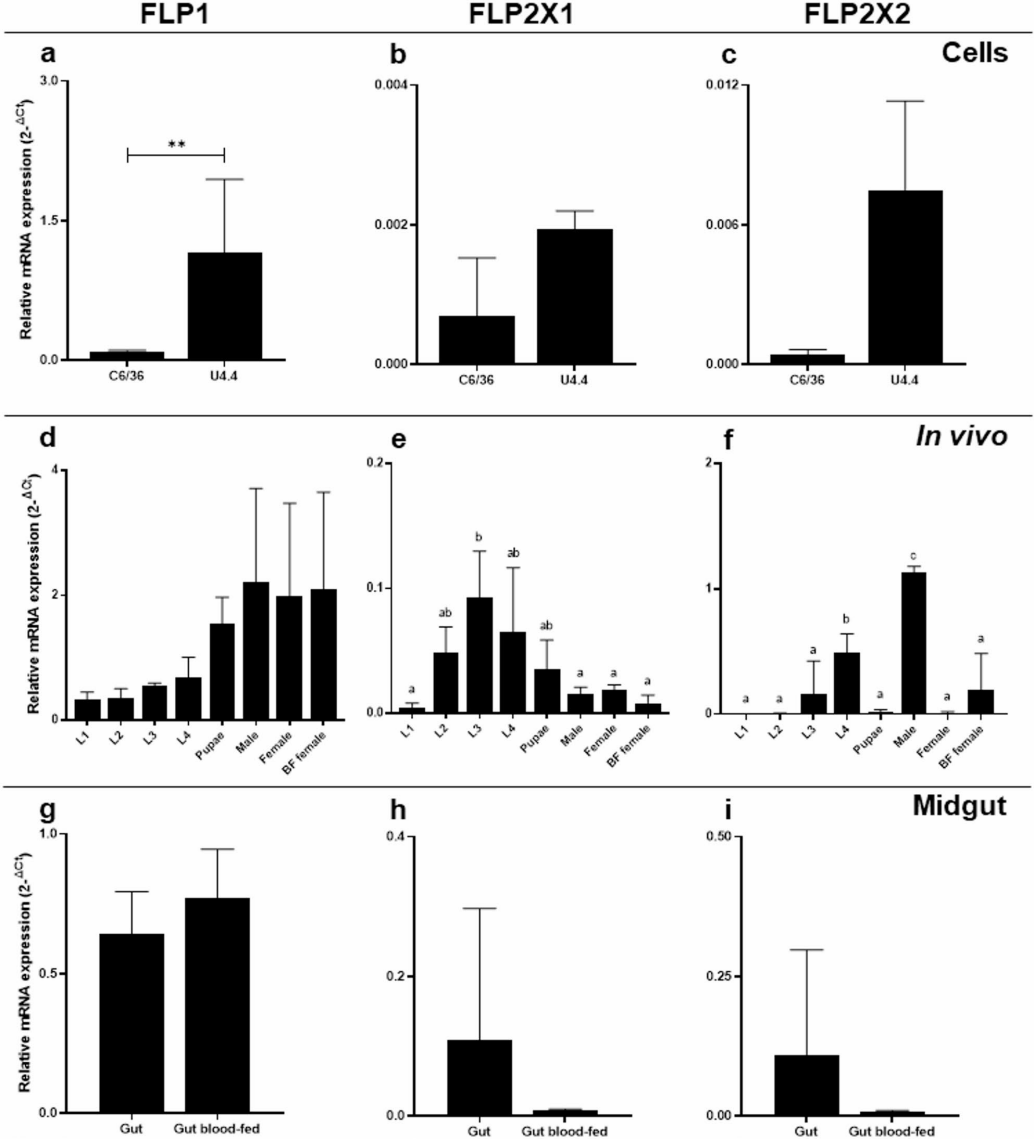
Relative mRNA levels of the furin encoding genes FLP1 (**a**,** d**,** g**), FLP2X1 (**b**,** e**,** h**), and FLP2X 2 (**c**,** f**,** i**) by RT-qPCR in *Ae. albopictus* cell lines, all life stages, and midgut samples. Transcripts of the housekeeping gene protein phosphatase-2 A (PP2A) were used for normalization and data were analyzed via ${2}^{-\varDelta{C}_{t}}$. Graphs show the mean relative expression (*n* = 3) and standard deviation (error bar) per sample. Statistical significance was determined by unpaired t-test (**p < 0.005) and one-way ANOVA and Tukey’s multiple comparison test. Different letters indicate statistical significance (*p* < 0.05)

Based on the putative furin homologs in *Ae. albopictus*, we designed specific primers targeting these genes and determined their mRNA expression in C6/36 and U4.4 cells, all four larval stages, pupae, males, females, blood-fed females, and midguts of non-blood fed and blood-fed females via RT-qPCR. FLP1 was the gene that was expressed consistently among the cells, tissue and all life stages, showing no significant differences among them (Fig. 2d). It showed its highest expression in pupal and adult stages, whereas the lowest expression was found in C6/36 cells (Fig. 2a). Interestingly, its expression in the midguts of non-blood fed and blood-fed females (Fig. 2g) was similar. The FLP2X1 expression was highest in the larval stages (Fig. 2e), except for the larval stage L1. No significant differences were found between the cells (Fig. 2b) and the midgut samples (Fig. 2h). FLPX 2 had the lowest overall expression of the three genes (Fig. 2f). We found the highest expression in males, while a low expression was observed in early larval stages (L1-L2). In both cell lines, the isoform was hardly expressed (Fig. 2c) similar to the midgut samples (Fig. 2i).

### Furin inhibitors

In previous studies, we developed the furin inhibitors MI-1148 [28], MI-1554 [27], and MI-1851 [25], which have been extensively characterized for their antiviral potency in mammalian cell cultures and for their toxicity in mice. These inhibitors have shown to be effective in inhibiting virus replication of highly pathogenic avian influenza viruses and arboviruses. Due to the amount of available data on these three inhibitors, they were selected for testing. Additionally, we synthesized the FITC-tagged furin inhibitor MI-1190 to study the uptake in aedine cell culture and in different developmental stages of *Ae. albopictus*. In order to retain the basic residues in the P1, P2, P4, and P5 positions, which are important for the inhibitory potency, the fluorophore FITC was coupled to the side chain of a lysine in P3 position via a β-alanine spacer. From the coupling of the FITC onwards, all reactions and the storage of the inhibitor were carried out under light protection, as FITC is prone to photobleaching. For inhibitor MI-1190, a *K*_i_ value of 500 pM was determined for human furin (Table 2, Fig. S1a), which is substantially higher those of the other inhibitors in this study. In contrast, the precursor 4-GMe-Phac-Arg-Lys(H-β-Ala)-Arg-4-Amba (MI-1189) lacking the bulky FITC label inhibits furin with a *K*_i_ value of 22.15 pM (Table 2, Fig. S1b), which is in a similar range as determined for inhibitors MI-1148, MI-1554, and MI-1851. The sequences, *K*_i_ values, and toxicity data in mice of the furin inhibitors used in this study are summarized in Table 2.

**Table 2:** Sequences, inhibitions constants, toxicity data of furin inhibitors used in this study

Furin inhibitor	Sequence	*K*_i_ value hfurin (pM)	Tolerated i.*p*. dose in mice (mg/kg)	Reference
MI-1148	4-GMe-Phac-Arg-Tle-Arg-Amba ×4 HCl	5.5	2.5	[28]
MI-1554	4-GMe-Phac-Arg-Tle-Lys-Amba ×4 HCl	8.8	5	[27]
MI-1851	4-GMe-Phac-Cav-Tle-Cav-Amba ×4 HCl	10.1	15*	[25]
MI-1189	4-GMe-Phac-Arg-Lys(H-β-Ala)-Arg-Amba ×4 TFA	22.15	no data	this study
MI-1190	4-GMe-Phac-Arg-Lys(FITC-β-Ala)-Arg-Amba ×4 TFA	500	no data	this study

*Not tested at higher concentrations

### Fluorescence microscopy with inhibitor MI-1190 in cell culture

**Fig. 3:**
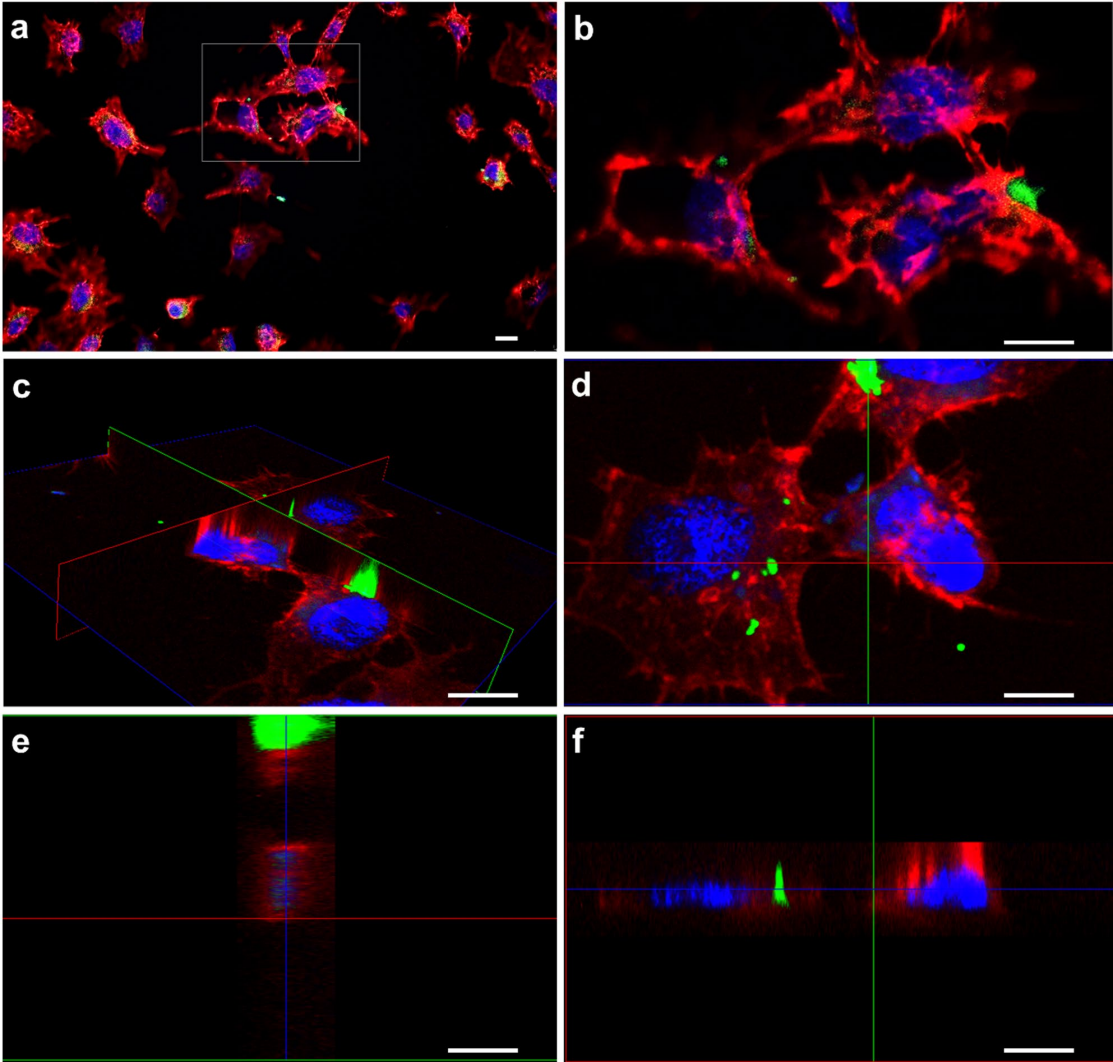
Confocal microscopy of the uptake and localization studies of the FITC-tagged inhibitor MI-1190 (green, stock 5 mM in 1:1 (v/v) H_2_O/DMSO), 50 µM final concentration) in C6/36 cells. Cells were counterstained with DAPI (blue) and phalloidin (red) to facilitate the localization of the furin inhibitor. (**a**): Overview at 200× resolution. (**b**): Zoom-in of the grey box of panel (**a**); (**c**): Oblique view on stained cells. (**d**): Top view of C. (**e**): Cross section of C. (**f**): Cross section of C. (**c-f**): Pictures were cross sectioned by the LASX software. Three cross sections are colored in either green, red or blue. Pictures (**d**), (**e**) and (**f**) show (c) from a different angle. Z-sectioning and stacking (1 μm slices) was done automatically and annotated by the LASX software. A representative image of this experiment is depicted in this figure. Scale bar: 50 μm

The visualization of the inhibitor uptake in C6/36 cells was facilitated by using the FITC-tagged compound MI-1190. Due to limited compound availability, the uptake was studied only in C6/36 cells. The images of the uptake and localization study were taken with a confocal microscope while co-localization images were taken with a DM5000B Leica fluorescence microscope. Images were analyzed with the LASX software. For confocal analysis, nuclei were counterstained with DAPI and membranes were labeled with phalloidin to facilitate the localization of the furin inhibitor. Using Z stacking with cross sections, we observed the green signal of our FITC-tagged inhibitor within the cells. Cross sections of chosen cells confirmed a distribution of the furin inhibitor among the cells (Fig. 3).

**Fig. 4:**
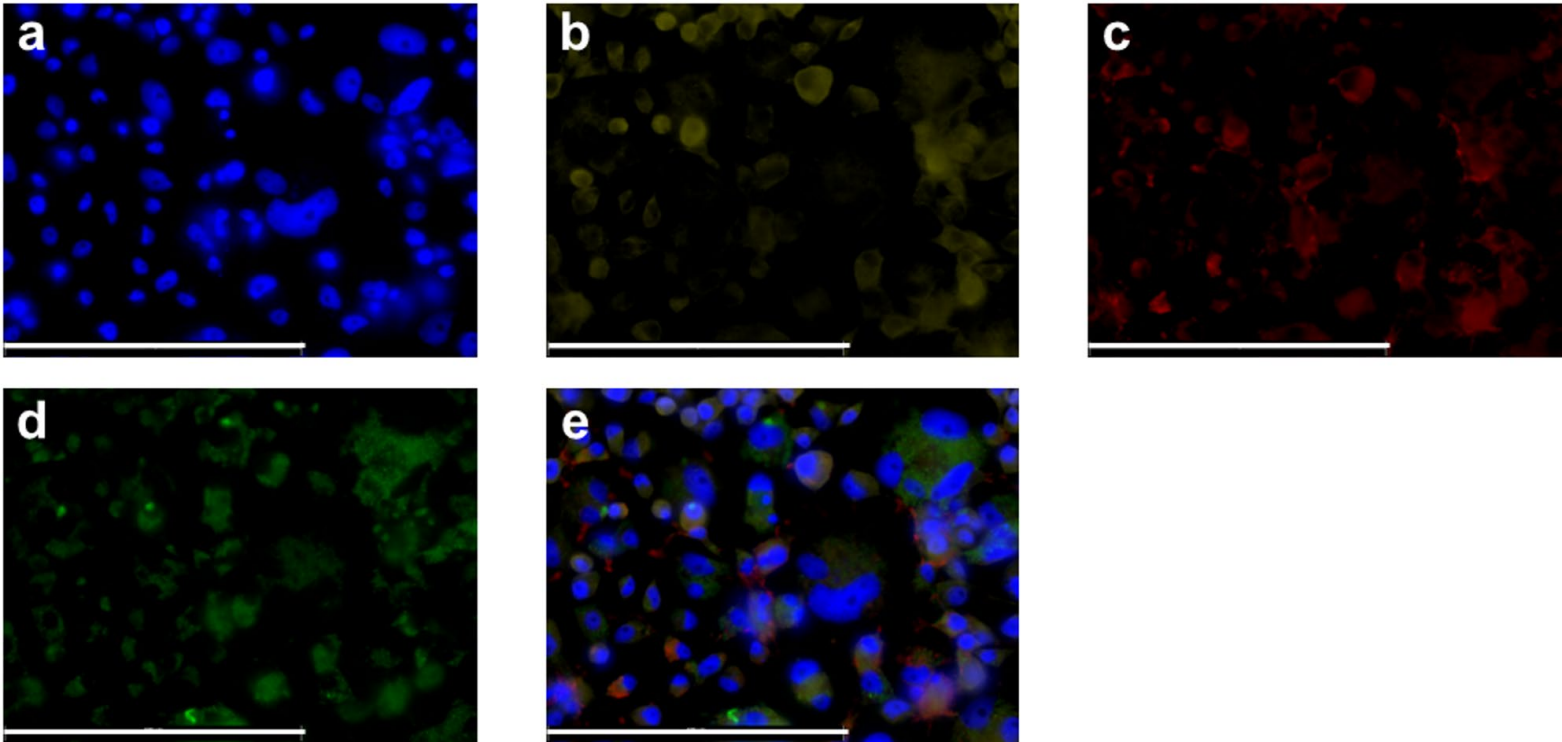
Immunofluorescence microscopy of the co-localization of the FITC-tagged inhibitor MI-1190 (green, stock 5 mM in 1:1 (v/v) H_2_O/DMSO), 10 µM final concentration), Golgi network (yellow), and the viral non-structural protein 1 in C6/36 cells. Cells were counterstained with DAPI (blue). (**a**): DAPI. (**b**): Golgin245. (**c**): non-structural protein 1 of SFV. (**d**): MI-1190. (**e**) Overlay. A representative image of this experiment is depicted in this figure. Scale bar: 100 μm

Additionally, to further assess the co-localization of MI-1190 with viral proteins, we performed a co-localization study in SFV4-infected C6/36 cells using a Golgi network marker. Co-localization of the viral non-structural protein 1 and the inhibitor within the Golgi network was observed (Fig. 4).

### Fluorescence microscopy with inhibitor MI-1190 in larvae

**Fig. 5:**
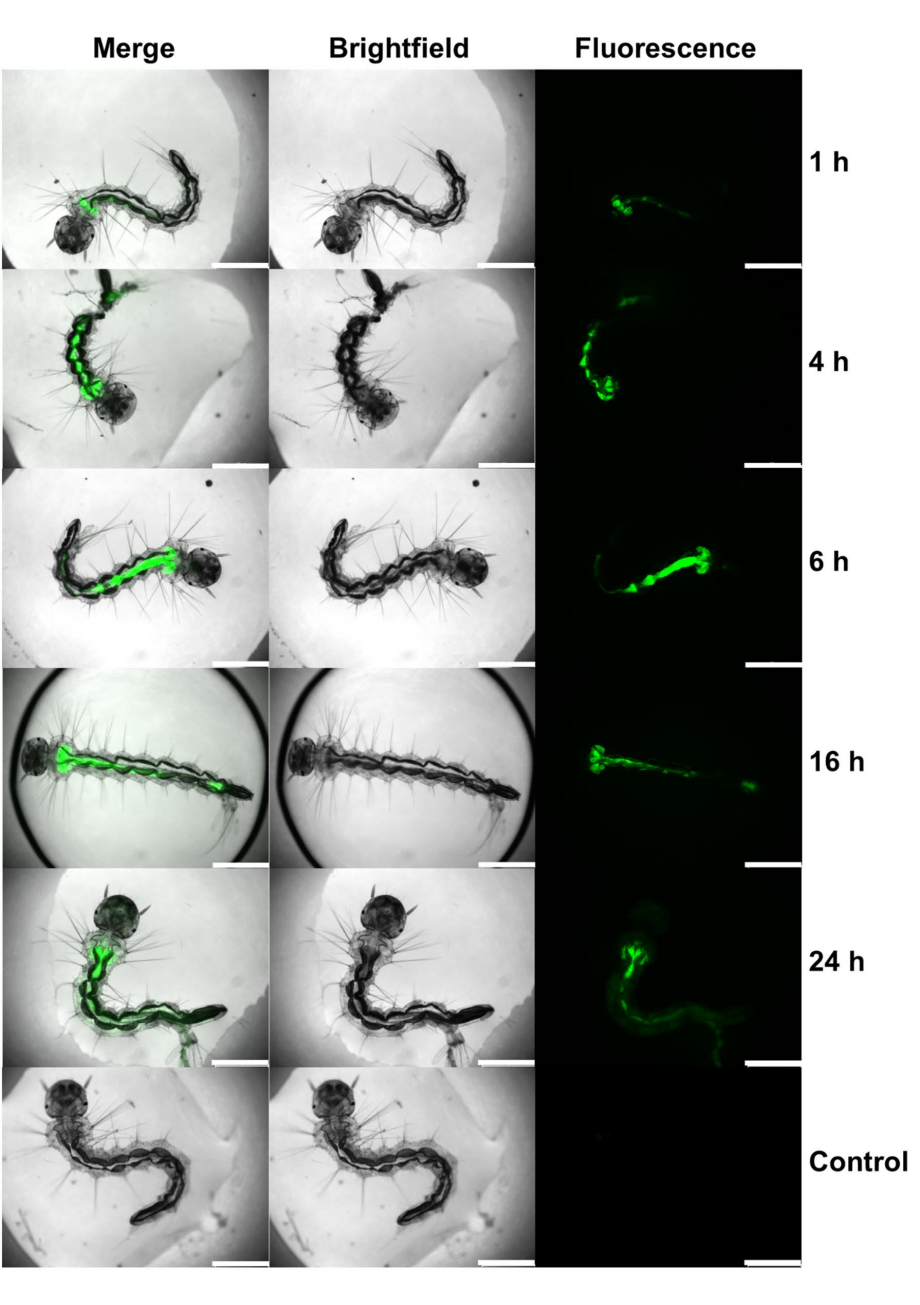
Uptake of the FITC-tagged inhibitor MI-1190 (stock 5 mM in 1:1 (v/v) H_2_O/DMSO, 100 µM per well) in *Aedes albopictus* stages L1-L2. Fluorescence microscopy was performed to observe the distribution of the inhibitor in larvae after constant exposure at 1, 4, 6, 16, and 24 h. Larvae were washed twice, trapped in 2 µL water during microscopy, then incubated again in the inhibitor-containing solution or in water (control), respectively. A representative image of the experiment is shown here. Scale bar: 20 μm

Larvae of *Ae. albopictus* in stages L1 and L2 were exposed to the FITC-tagged inhibitor MI-1190 for different times (1, 4, 6, 16, and 24 h). Images of the larvae were taken with a fluorescence microscope using a GFP filter in combination with a brightfield filter. The compound first accumulated in the gastric caeca, then in the digestive tract, spreading throughout the entire digestive system before finally reaching the cloaca. After 24 h, images suggested distribution throughout the whole larva, except for the head (Fig. 5). The control group was placed in water and no fluorescence signal was detected in their tissues. No toxic effects were observed during the duration of the experiment.

### Fluorescence microscopy with inhibitor MI-1190 in *Ae. albopictus* female mosquitoes

**Fig. 6:**
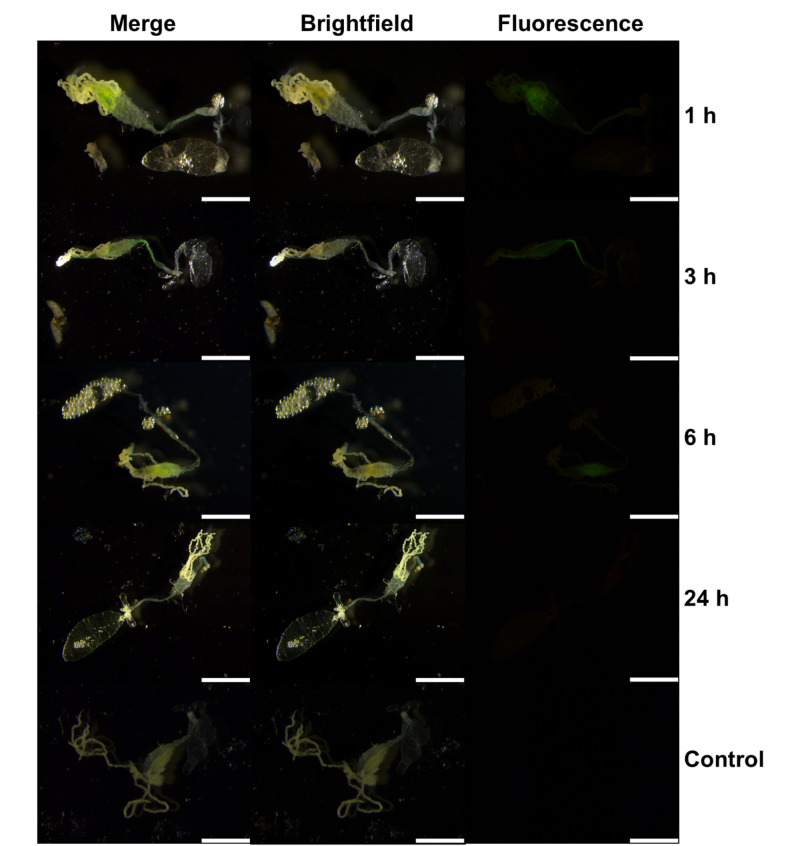
Distribution of the FITC-tagged inhibitor MI-1190 (stock 5 mM in 1:1 (v/v) H_2_O/DMSO, final concentration 1 mM) in females of *Ae. albopictus* after microinjection (volume: 101.2 nL). Females were anesthetized using CO_2_, passed through ethanol, and dissected in PBS. Fluorescence microscopy was performed to observe the distribution of the inhibitor in females after microinjection at 1, 3, 6, and 24 h post-treatment. The control group was injected with water. A representative image of the experiment is shown here. Scale bar: 5 mm

Five to ten day old females of *Ae. albopictus* were used to observe the distribution of the FITC-tagged inhibitor MI-1190 after microinjection over time. The injected females were dissected and images were captured with a DM5000B Leica fluorescence microscope using a GFP filter in combination with a brightfield filter. At 1 hpt, the compound accumulated in the gut but was not detected in the rest of the body. At 3 and 6 hpt, we observed a decrease in fluorescence. The inhibitor exhibited limited permeability across the intestinal epithelium, indicating restricted diffusion through the gut membrane (Fig. 6). Finally, no fluorescence signal was visible at 24 hpt, indicating complete clearance of the compound. The control group was injected with water. No fluorescence was detected at any time point.

### Cytotoxicity in cell culture

**Fig. 7:**
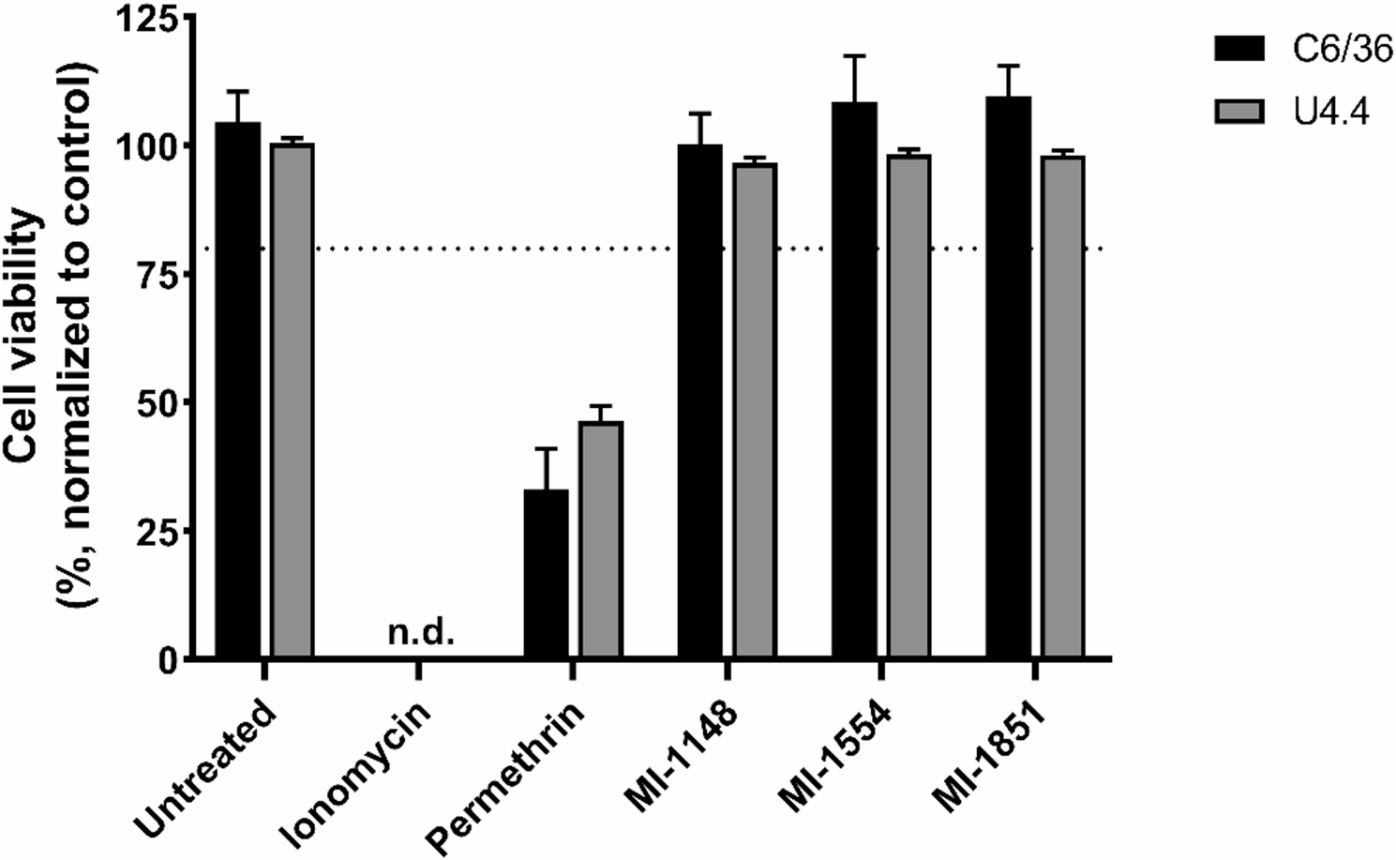
Cell viability of C6/36 and U4.4 cells in the presence and absence of the furin inhibitors. Confluent cells were treated with the indicated compounds (stock solution: 10 mM in water, 100 µM per well). Ionomycin and permethrin were used as positive controls. At 48 h the cell viability was assessed via ATP quantification by CellTiter-Glo assay. The cell viability was normalized to the water control and expressed as a percentage (%). The mean cell viability (*n* = 5) is shown and the error bars represent standard deviation (%). The dotted line represents the toxicity cut-off set at 80%. n.d.= not detectable

Furin inhibitors were tested in the aedine cell lines C6/36 and U4.4 for cytotoxicity by quantifying the ATP levels of the different treatments using the CellTiter-Glo assay. Confluent cells were treated with 100 µM of the furin inhibitors or ionomycin as a positive control for cytotoxicity for 48 h. Permethrin was included as an insecticide with mosquitocidal activity. The cell viability was normalized to the water treated samples, which served as the vehicle control, and expressed as a percentage. No cytotoxic effects (cell viability > 80%) were observed for the inhibitors MI-1148, MI-1554, and MI-1851 (Fig. 7). Therefore, we used a concentration of 100 µM in all further experiments. As expected, ionomycin and permethrin showed strong cytotoxic effects.

### Toxicity in *Ae. albopictus* larvae

**Fig. 8:**
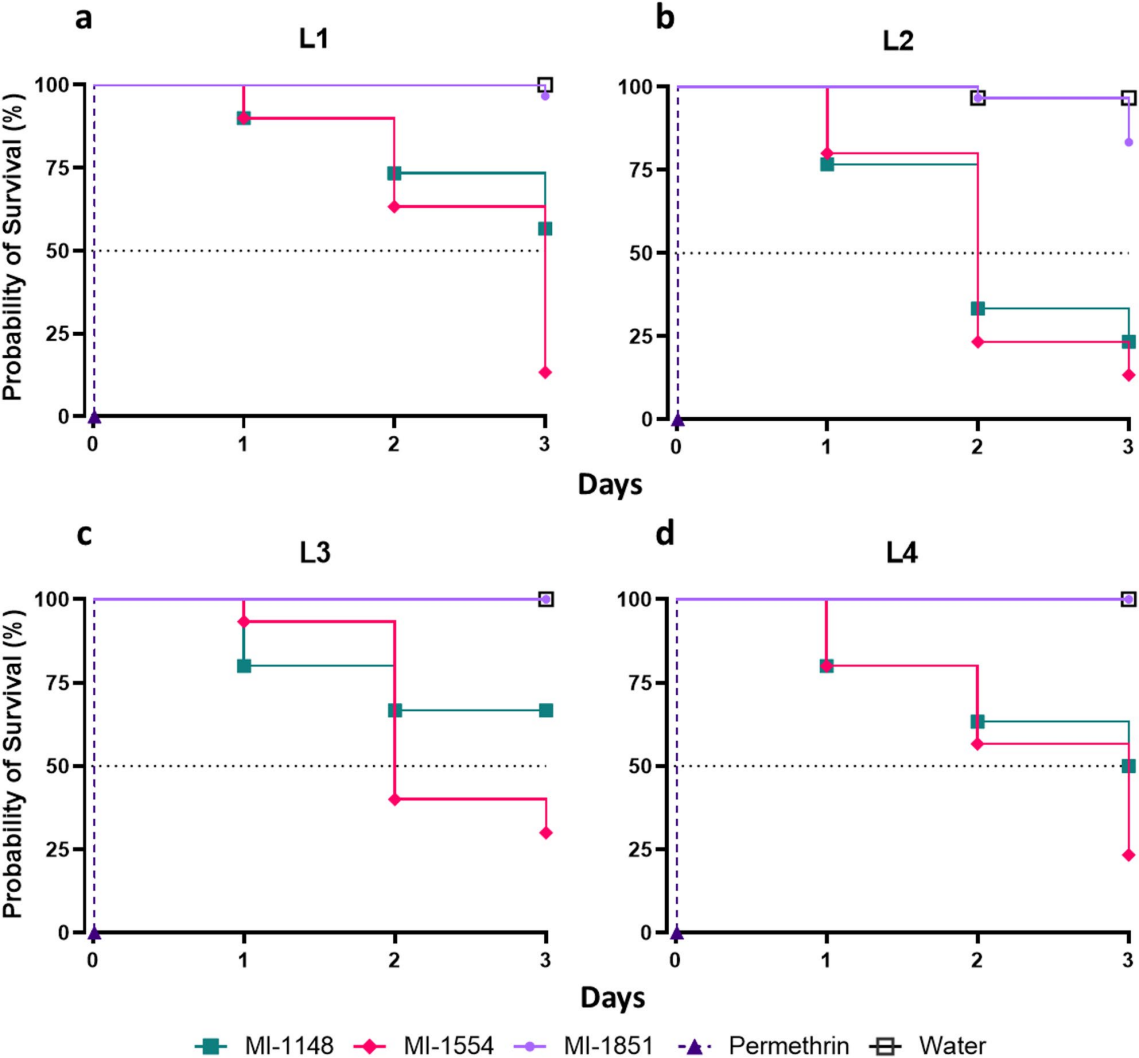
Toxicity of furin inhibitors in *Ae. albopictus* larval stage (**a**) L1, (**b**) L2, (**c**) L3, and (**d**) L4 displayed as Kaplan-Meier plot. Furin inhibitors MI-1148, MI-1554, and MI-1851 as well as the insecticide permethrin were added to the breeding water of the larvae (stock solution: 10 mM, 100 µM final concentration in the assay). Untreated larvae in water were used as negative control. The dotted line represents the 50% mortality cut. All treatments were compared to the water control. Statistical analysis (Kaplan-Meier) indicated significant differences (*p* < 0.0001) for the treatments with MI-1148, MI-1554, and permethrin (*n* = 30 per treatment)

After completing the evaluation of the furin inhibitors in cell culture, we proceeded to test them in vivo starting with the four larval stages (L1-L4). The inhibitors were added to the rearing water of the mosquito larvae at the same concentration used in the cell assays. Inhibitor MI-1851 showed limited toxicity at all larval stages, with a probability of survival comparable to untreated controls (Fig. 8). In contrast, inhibitors MI-1148 and MI-1554 showed clear toxic effects and significantly reduced the probability of survival. Inhibitor MI-1554 was the most toxic compound, reducing the probability of survival to < 30%, whereas MI-1148 was less toxic, reducing the probability of survival to 20–70% depending on the larval stage. For comparison, permethrin at the same concentration as the furin inhibitors was lethal to all larvae on day 1 at all developmental stages.

### Toxicity in female *Ae. albopictus*

**Fig. 9:**
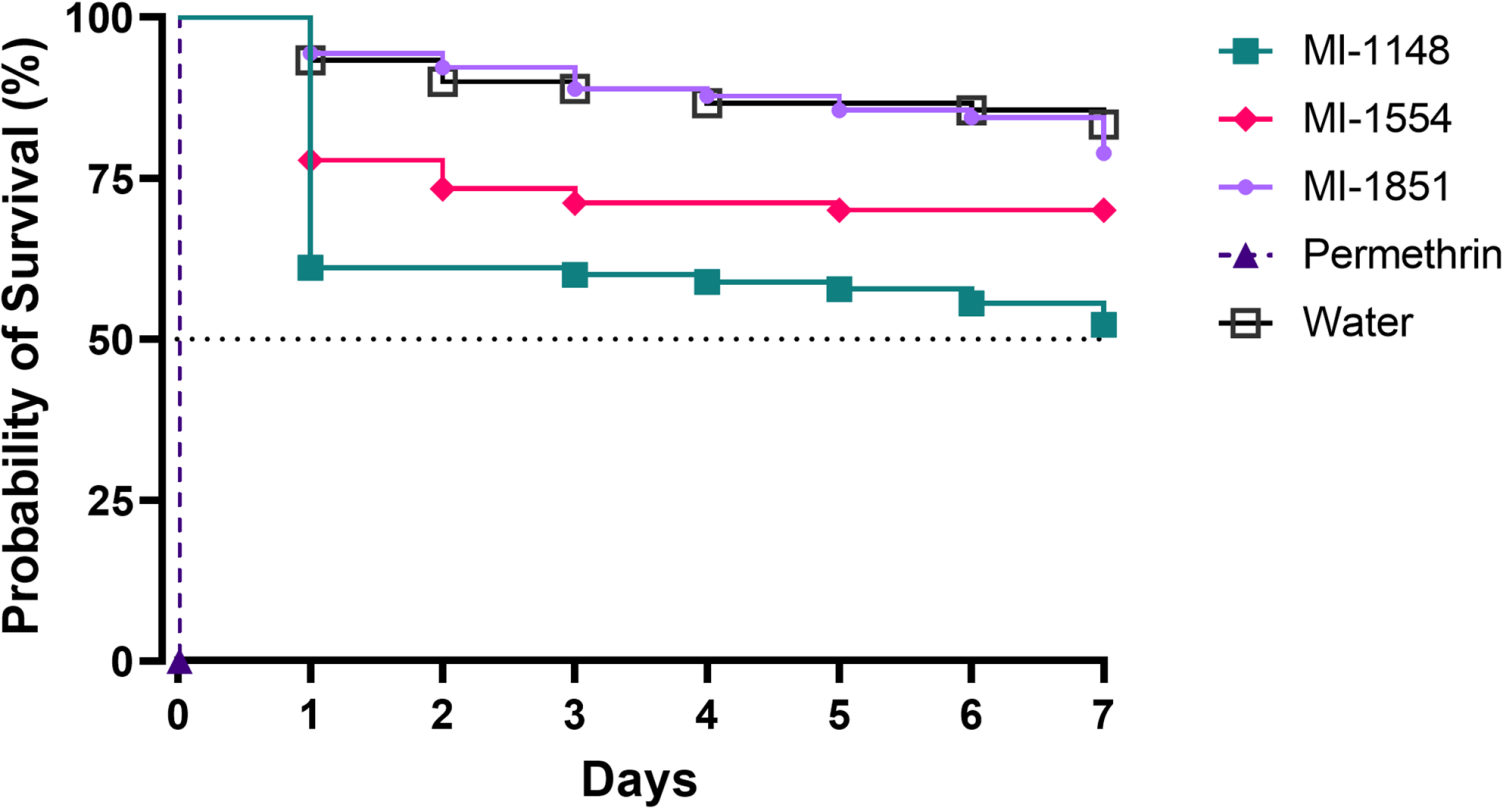
Toxicity of furin inhibitors in *Ae. albopictus* female mosquitoes. Compounds MI-1148, MI-1554, and MI-1851 were microinjected into the mesokatepisternum of the females. Permethrin and water-treated females were used as controls. A total volume of 101.2 nL of a 1 mM stock solution in water was injected into females of each treatment group (*n* = 90). The dotted line represents the 50% mortality cut. All treatments were compared to the water control. Statistical analysis (Kaplan-Meier) indicated significant differences for the treatments with MI-1148 and permethrin (*p* < 0.0001) as well as the treatment with MI-1554 (*p* < 0.01)

To test the toxicity of the furin inhibitors in female mosquitoes, they were microinjected with a total volume of 101.2 nL (50.6 nL twice) of a 1 mM stock solution of each inhibitor. Consistent with the results of the larval experiments, inhibitor MI-1851 was the least toxic compound leading to a survival of approximately 80%, which is very similar to the negative control, where only water was injected (~ 83% survival). In contrast, inhibitor MI-1148 reduced the probability of survival to 50% and MI-1554 to 75% (Fig. 9). Permethrin was lethal immediately after injection in all tested females. Furthermore, we evaluated the toxicity of MI-1851 in a feeding approach with erythrocytes in a four-day assay. No mortality was recorded by the end of the experiment (Fig. S3).

### Effects of furin inhibitors on SFV encoded mCherry expression in aedine culture

**Fig. 10:**
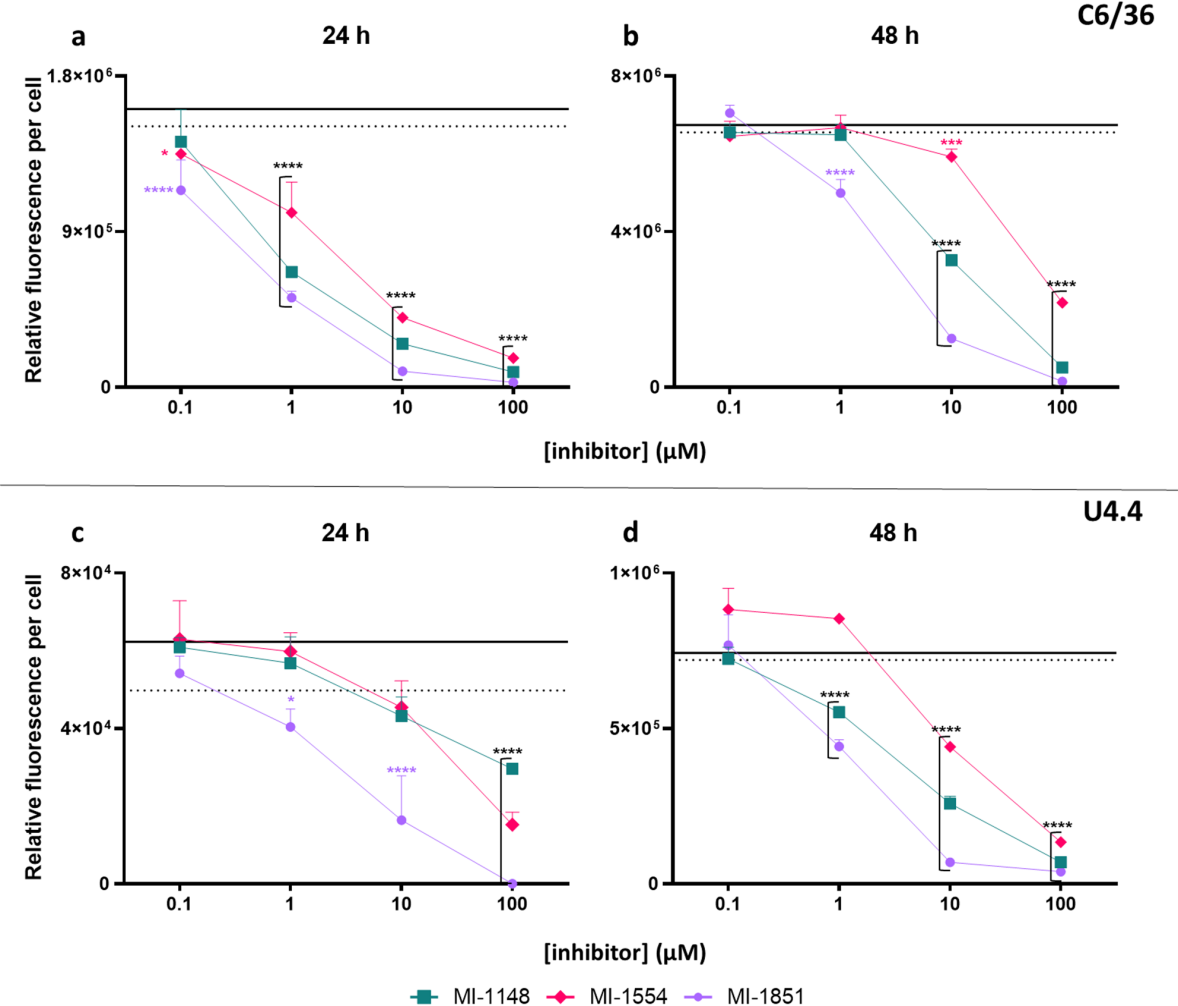
Antiviral efficacy of the furin inhibitors upon mCherry-Semliki Forest virus (SFV) infection in (**a**,** b**) C6/36 and (**c, d**) U4.4 cells at 24 and 48 h post-infection. Cells were infected with SFV-mCherry (MOI: 0.01) for 1 h. Furin inhibitors MI-1148, MI-1554, and MI-1851 were added after infection at concentrations of 0.1, 1, 10, and 100 µM. The data are mean values (*n* = 3) of the relative fluorescence per cell (total red intensity/total cell count) and the error bars represent standard deviations. The dotted line represents the mean relative fluorescence per cell of SFV. The solid line represents the water control. Significance level of inhibition were made between SFV and the inhibitor treatment groups: *p < 0.05; **p < 0.005; ***p < 0.001; ****p < 0.0001

Since we observed no cytotoxic effects on the cells by the furin inhibitors, we proceeded to test their antiviral efficacy. Aedine C6/36 and U4.4 cells were grown to confluency and subsequently infected with SFV-mCherry at a MOI of 0.01 for 1 h. Following infection, the cells were treated with the furin inhibitors MI-1148, MI-1554, and MI-1851 at concentrations of 100, 10, 1, and 0.1 µM. Water was used as a negative control. The relative fluorescence per cell was measured at 24 and 48 hpi. Across both cell lines and time points, we observed a significant dose-dependent inhibition by each inhibitor (Fig. 10). At the highest concentration of 100 µM, all inhibitors significantly reduced the SFV encoded mCherry expression in both cell lines at 24 hpi. In C6/36, the antiviral effect of the furin inhibitors was more pronounced within the first 24 hpi (Fig. 10a). At 48 hpi, a concentration of 1 µM was no longer effective with the only exception of MI-1851 (Fig. 10b). In contrast, MI-1148 and MI-1554 treatments below a concentration of 100 µM in U4.4 cells showed no or only little inhibition of the SFV encoded mCherry expression (Fig. 10c). However, at 48 hpi, the protective effect of the inhibitors was observed at a concentration of 1 µM in MI-1148 and MI-1851 and at 10 µM in MI-1554 (Fig. 10d). Among the inhibitors, MI-1851 showed the highest protection, being effective at concentrations up to 1 µM in both cell lines up to 48 hpi.

Comparable results were obtained with the non-tagged SFV4 wild-type virus in C6/36 cells (see Fig. S7), where infectivity was assessed by a TCID₅₀ assay rather than by reporter readout. Furthermore, Western blot analysis of SFV4-infected C6/36 and BHK-21 cells revealed that the viral E3-E2 protein remained uncleaved in the presence of the inhibitor MI-1148 (see Fig. S8). In contrast, untreated infected cells displayed the expected processing of E3 to E2, indicating that MI-1148 effectively blocks furin-mediated cleavage.

### Effect on SFV encoded mCherry expression in vivo

The first approach for in vivo infection was tested in L3-L4 larvae by feeding them on previously infected C6/36 cells or allowing them to swim in a pool of SFV. Feeding the larvae on infected cells with SFV-mCherry initially led to a fluorescent signal in the larval guts within the first 24 h. However, the signal faded after one day (Fig. S4). No SFV encoded mCherry reporter expression could be determined for any larval sample after that time point and therefore, no further assessment with the furin inhibitors was carried out.

**Fig. 11:**
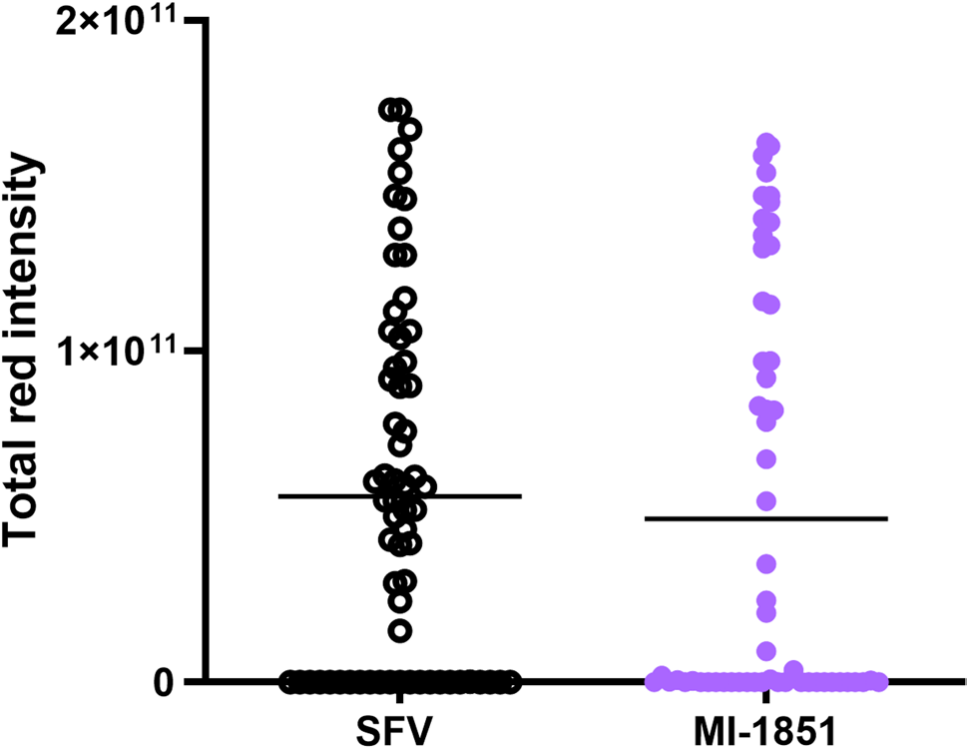
Antiviral activity of inhibitor MI-1851 in females infected with mCherry-Semliki Forest virus (SFV), assessed in C6/36 cells at 48 h post-infection. Cells were infected with the supernatant from disrupted, engorged females either infected with SFV-mCherry in presence of the inhibitor MI-1851 (*n* = 57) or with SFV-mCherry alone (*n* = 65) for 1 h. Virus spread was quantified as the total red intensity from the infected cells. The background signal was removed using the signal of untreated cells. Data points represent the mean integrated fluorescence from single samples. Statistical analysis was carried out by Mann-Whitney test (*p* = 0.658)

Based on the results obtained in the female mosquitoes’ toxicity assay, we decided to use only compound MI-1851 for the antiviral efficacy assay in females. Females were offered an infected blood meal with SFV-mCherry in the presence or absence of inhibitor MI-1851 (100 µM). After 3 days, the mosquitoes were individually disrupted and the samples were used to infect C6/36 cells. At 48 hpi, the mean total red intensity of the treatment group treated with MI-1851 was slightly lower (11%) compared to the SFV control group. However, no significant difference was found between the SFV control and the MI-1851 treatment (*p* = 0.658, Fig. 11). Similarly, the infection rates observed in the treatment group (64.9%) and the control group (66.2%) did not differ significantly.

## Discussion

Arboviral diseases are expanding, emerging, and re-emerging worldwide. Despite this, no approved antiviral treatments exist for the vast majority of these diseases and vaccines remain unavailable or limited. This persistent gap in therapeutic options highlights the necessity of developing strategies that complement traditional vector control methods. Suppression of arbovirus replication through antiviral compounds within the mosquito vector by targeting conserved cellular factors remains a largely unexplored approach that could improve these strategies. One promising target is furin, a host proprotein convertase involved in the proteolytic activation of many viral glycoproteins, including those of alphaviruses [47, 48]. This study aimed to characterize furin homologs in the mosquito *Ae. albopictus* and evaluate furin inhibitors as a complementary strategy to vector control and disruption of arbovirus transmission.

The identification of *Ae. albopictus* furin-like proteins FLP1 and the two isoforms FLP2X1 and FLP2X2 aligns with previous findings in other dipteran species such as *Ae. aegypti* and *D. melanogaster* [17, 19, 20]. This highlights the evolutionary conservation of this protease and thus, its potential as a broad-spectrum host factor for virus replication among different invertebrate and vertebrate hosts. The distribution of these furin-like proteases across cell lines and life-cycle stages underscores the need for stage-specific targeting. FLP1 was consistently expressed throughout all stages, having its highest expression in pupae and adult stages. Of the three identified proteases, FLP1 may be the most relevant target for inhibiting virus replication. The furin homologs of *Ae. aegypti* and *D. melanogaster*, Fur1 and Dfur1 respectively, have been linked to the vitellogenin convertase [20, 21]. The notable shared identity between these two homologs and FLP1 (Table S2), suggests a close relation between the proteases. Understanding the expression levels of relevant furin-like proteases is crucial for optimizing antiviral strategies. This is especially important for compounds like furin inhibitors, whose effectiveness depends on the presence and activity of host furin-like proteases.

In cell culture, uptake and intracellular distribution of compounds can be investigated using several methodological approaches. A commonly employed strategy is to label the compounds, thereby enabling the tracking of their cellular entry and localization. Radiolabeling with isotopes such as ^3^H or ^14^C allows highly accurate quantification of uptake through radioactivity measurements. Here, the native compounds can be directly used in experimental assays. However, experiments must be conducted in designated radioisotope laboratories. As an alternative, fluorescent dyes such as FITC, TRITC, or rhodamine can be conjugated to the compounds, permitting visualization of their intracellular distribution by fluorescence microscopy. This approach requires covalent attachment of the fluorophore, which inevitably modifies physicochemical properties such as molecular weight and polarity. FITC represents one of the smallest fluorescent moieties and we therefore anticipated that the cellular uptake and subcellular localization of the FITC-conjugated compound closely resembles those for the corresponding non-labeled inhibitors. Therefore, we synthesized the FITC-tagged furin inhibitor MI-1190. The *K*_i_ value of this inhibitor is 500 pM, which is approximately 100-fold higher than MI-1148, MI-1554, and MI-1851. The decrease of inhibitory efficacy is most likely caused by the introduction of the bulky FITC label. However, inhibitor MI-1190 remained a very potent furin inhibitor. An initial visualization step was essential for determining compound-delivery strategies based on uptake and stability, as these factors influence the compounds’s ability to inhibit virus replication. In mammals, furin activity is reported to occur in the trans-Golgi network [13]. Consistent with this localization, MI-1190 was detected near the nucleus in C6/36 cells (Fig. 3) and showed overlap with the viral non-structural protein 1 as well as the Golgi marker Golgin245 (Fig. 4). In mosquitoes, arboviruses must overcome tissue barriers before they can become systemically established [49]. Here, the midgut plays a key role in determining whether an arbovirus can successfully infect and spread within the mosquito [50]. While some viruses fail to infect due to receptor mismatch or immune responses, others replicate in the midgut but do not reach other tissues [49]. This makes the midgut a strategic site for blocking virus transmission. The FITC-tagged furin inhibitor MI-1190 was used for visualization assays. No mortality was observed in either the larvae or adult females. In our experiments, the larvae were continuously exposed to the inhibitor. The uptake was detectable after one hour, and the compound accumulated in the digestive tract (Fig. 5). In contrast, in female mosquitoes, we dissected the animals after administering the compound via microinjection, as their dark pigmentation prevented clear visualization of the inhibitor. Fluorescence faded within a day of injection, suggesting enzymatic degradation or excretion of the compound (Fig. 6). Based on these findings, inhibitor MI-1851 was administered through the infectious blood meal and maintained throughout the antiviral experiment. This approach ensured early and sustained availability of the compound without harming the mosquitoes.

Having established that the furin inhibitors were taken up in cells and located in larvae and female mosquitoes, we assessed the toxicity of the previously described furin inhibitors MI-1148, MI-1554, and MI-1851 to ensure their suitability for antiviral application. No toxic effects of the furin inhibitors were observed at the tested concentration in C6/36 and U4.4 cells (Fig. 7). These results were consistent with previous assays in mammalian cells, where MI-1148 also showed no toxicity at 100 µM [51]. The derivatives MI-1554 and MI-1851 were tested at a maximum concentration of 50 µM before, but demonstrated similar safety profiles [25]. In contrast to the results in cell culture, the inhibitors MI-1148 and MI-1554 showed strong toxic effects across all larval stages (Fig. 8) and female mosquitoes (Fig. 9). The strong multibasic character of MI-1148, with its three guanidino groups and the benzamidine in the P1 position, is likely the cause of the high toxicity of this compound. In order to reduce the overall basicity of this compound, in MI-1554 the Arg P2 was replaced by Lys. This change slightly decreased its potency but also was less toxic in mice [27]. Due to the toxicity observed for both inhibitors, they were excluded from the in vivo antiviral assays. In contrast, MI-1851 had previously exhibited a more favorable safety profile. The substitution of Arg P2 and P4 by Cav significantly reduced its toxicity in mice, while the antiviral potency remained strong [25]. The exact mechanism of its reduced toxicity remains unclear. However, it is hypothesized that Cav is only partially charged in the extracellular space and during circulation. This could allow for more efficient uptake and transport compared to the fully protonated inhibitor MI-1148. At the site of action, in the slightly acidic trans Golgi network, both inhibitors are fully protonated in order to efficiently inhibit furin. Although we observed the antiviral effect in mosquito cell lines treated with the furin inhibitors, the direct inhibition of *Ae. albopictus* FLP1 or FLP2 remains to be demonstrated. Therefore, it is possible that other proteases contribute to the viral E2E3 cleavage. In mammals, furin is one of nine proprotein convertases (PCs), several of which recognize multibasic motifs depending on their localization and tissue-specific expression [52]. Compensatory activity among PCs has been reported and additional trypsin-like proteases, such as TMPRSS13, can also cleave multibasic sites [53]. For SFV, the p62 cleavage motif (_330_RHRR) has thus far been attributed only to furin [48], similarly to the SINV p62 site (_325_RSKR) [54]. However, the CHIKV p62 cleavage site (_322_RQRR) can be processed not only by furin but also by PACE4 and PC5 [55].

All three inhibitors displayed potent antiviral activity against SFV encoded mCherry expression at concentrations up to 10 µM in C6/36 and U4.4 cells (Fig. 10). Most interestingly, MI-1851 remained effective even at 1 µM after 48 hpi in both cell lines. The effectiveness in blocking SFV encoded mCherry reporter expression by furin inhibitors in mosquito cells was similar to that previously described in mammalian cells. The inhibitor MI-1148 had previously exhibited potent antiviral activity against SFV4 and CHIKV in mammalian BHK-21 cells at 25 µM [29]. Similarly, the inhibitors MI-1554 and MI-1148 also effectively reduced West Nile virus and DENV-2 replication in Huh-7 cells at 50 µM [27, 31], and MI-1851 but at 16 µM [25]. Interestingly, SFV-mCherry exhibited faster and higher fluorescence signals in C6/36 cells compared to U4.4 cells, though the inhibitory effect of the compounds remained present at both time points. In contrast to the U4.4 cell line, C6/36 cells have a dysfunctional RNAi response that allows for high viral titer production [56, 57]. This viral replication pattern has also been observed previously when comparing SFV encoded mCherry reporter signal expression in both cell lines while targeting the mCherry marker with dsRNA [58]. Although SFV-mCherry functioned as a stable and reliable surrogate for intracellular replication and translation in this study (passage two), we observed a gradual decrease in fluorescence intensity over successive passages. While SFV-mCherry expression remained detectable up to four passages (Fig. S5), the progressive decline in signal intensity indicates reporter attenuation over time. This highlights an inherent limitation of fluorescent reporter viruses that must be considered when using them for multi-cycle replication studies.

Despite the promising results obtained in vitro, the in vivo experiments faced notable limitations. In our attempts to infect *Ae. albopictus* larvae with SFV, either by swimming in a viral pool or by feeding with infected cells, we were unable to determine a virus signal. The absence of a confirmed infection in larvae limited our ability to test the antiviral potential of inhibitor MI-1851. Future studies could explore alternative arbovirus models and protocols like the ones used for Usutu virus [59] or DENV [60]. In adult female mosquitoes, the concomitant delivery of the inhibitor MI-1851 with an infectious blood meal exerted only a slight effect on the SFV encoded mCherry signal expression (Fig. 11). The limited efficacy observed in vivo may be attributable to variability in compound uptake, degradation, or limited distribution of the inhibitor. A previous study found that β-d-*N*^4^ hydroxycytidine (EIDD 1931) inhibited CHIKV replication in mosquito cells and ex vivo in *Ae. aegypti* guts. However, it failed to reduce infection when delivered via blood feeding. This finding is consistent with the results of our in vivo study, as the compound was rapidly cleared from the mosquito midgut [61]. In order to confirm the potential of the inhibitor MI-1851 against other arboviruses, such as DENV, ZIKV, and CHIKV, optimization of the delivery methods of the antiviral compounds and increased sample sizes are required.

Interestingly, the in vivo derived toxicity responses to furin inhibitions in mosquitoes mirrored those previously observed in mice [25]. This suggests that mosquitoes might serve as advantageous alternatives to mice for preliminary antiviral testing in numerous studies regarding ethical considerations, cost-effectiveness, and, due to their small size and simple rearing, also scalability [62]. This approach could substantially streamline preliminary evaluations of host-targeted antiviral strategies. However, the high conservation of furin between mammals and mosquitoes makes broad-spectrum furin inhibitors not an ideal alternative for field applications due to potential off-target effects. Other methods such as RNAi, gene drive, or CRISPR-Cas9 mediated gene knockdown could be used to specifically target mosquito furin and provide an alternative solution to fight the spread of arboviruses in their mosquito vectors.

Our results suggest that addressing furin for the suppression of arbovirus replication in mosquito could be a promising strategy, even though antiviral activity in vivo was limited under the conditions tested. This study represents a further step towards expanding the range of vector-based antiviral strategies. Further research needs to focus on exploring additional furin-targeting compounds, testing other arbovirus models, and refining the mosquito-based experimental methodologies.

## Supplementary Information

Supplementary Material 1

## Data Availability

The datasets used and/or analysed during the current study are available from the corresponding author on reasonable request.

## Abbreviations

DENV: Dengue virus
ZIKV: Zika virus
WNV: West Nile virus
CHIKV: Chikungunya virus
SFV: Semliki Forest virus
FLP1: Furin-like protease 1
FLP2: Furin-like protease 2
Ala: Alanine
Amba: 4-Amidinobenzylamide
Arg: Arginine
Boc: Tert-butyloxycarbonyl
Cav: Canavanine
Dde: 1-(4,4-Dimethyl-2,6-dioxocyclohex-1-ylidene)ethyl
DMF: Dimethylformamide
DMSO: Dimethylsulfoxide
FITC: Fluorescein isothiocyanate
Fmoc: 9-Fluorenylmethoxycarbonyl
GMe: 4-Guanidinomethyl
i.p.: Intraperitoneal
Lys: Lysine
Tle: Tert-leucine
TFA: Trifluoroacetic acid
Pbf: 2,2,4,6,7-Pentamethyldihydrobenzofuran-5-sulfonyl
Phac: Phenylacetyl
