# Correction: Furin as target for suppression of mosquito-borne viruses

**DOI:** 10.1186/s12985-026-03199-x

**Published:** 2026-05-28

**Authors:** Alejandra Centurión, Bodunrin Omokungbe, Markus Oberpaul, Ludwig Dersch, Sabrina Stiehler, Cross Chambers, Marcus Lechner, Tim Lüddecke, Alejandra Vilcinskas, Torsten Steinmetzer, Kornelia Hardes

**Affiliations:** 1 LOEWE Centre for Translational Biodiversity Genomics (LOEWE-TBG), Frankfurt am Main, Germany; 2 Department of Pest and vector insect control, Fraunhofer Institute for Molecular Biology and Applied Ecology IME, Branch of Bioresources, Giessen, Germany; 3 Department of Biodiversity, Fraunhofer Institute for Molecular Biology and Applied Ecology IME, Branch for Bioresources, Giessen, Germany; 4 Institute for Insect Biotechnology, Justus-Liebig University, Giessen, Germany; 5 BMBF Junior Research Group in Infection Research ASCRIBE, Giessen, Germany; 6 Center for Synthetic Microbiology (SYNMIKRO), University of Marburg, Marburg, Germany; 7 Institute of Pharmacology, University of Marburg, Marburg, Germany; 8 Institute of Pharmaceutical Chemistry, University of Marburg, Marburg, Germany

**Correction: Virology Journal (2026) 23:92**

10.1186/s12985-026-03127-z

In the sentence beginning ‘This ubiquitous type-I trans­membrane protein.....’ in the *Background* section of this article [1], the arrow (↓) was inadvertently converted into the letter “i”. The corrected sentence is: “This ubiquitous type-I transmembrane protein contains a Ca2+-dependent subtilisin-like serine protease domain and preferentially cleaves its substrates after the multibasic motif R-X-(R/K)-R↓-X [13].”

The original article has been corrected.
