# Supplementary material for: Furin as target for suppression of mosquito-borne viruses
Source: Virol J. 2026 Mar 11;23:92. doi: 10.1186/s12985-026-03127-z (PMC13063453; doi:10.1186/s12985-026-03127-z)
Supplement: Supplementary file 1 — Supplementary Material 1 [file 12985_2026_3127_MOESM1_ESM.docx]

**Furin as target for suppression of mosquito-borne viruses**

**Alejandra Centurión^1,2^, Bodunrin Omokungbe^1,2^, Markus Oberpaul^2,5^, Ludwig Dersch^1,3^, Sabrina Stiehler^4^, Cross Chambers^2,^ Marcus Lechner^6,7^, Tim Lüddecke^1,3^, Andreas Vilcinskas^1,2,4^, Torsten Steinmetzer^8^, Kornelia Hardes^1,2,5*^**

^1^ LOEWE Centre for Translational Biodiversity Genomics (LOEWE-TBG), Frankfurt am Main, Germany

^2^ Department of Pest and vector insect control, Fraunhofer Institute for Molecular Biology and Applied Ecology IME, Branch of Bioresources, Giessen, Germany

^3^ Department of Biodiversity, Fraunhofer Institute for Molecular Biology and Applied Ecology IME, Branch for Bioresources, Giessen, Germany

^4^ Institute for Insect Biotechnology, Justus-Liebig University, Giessen, Germany

^5^ BMBF Junior Research Group in Infection Research ASCRIBE, Giessen, Germany

^6^ Center for Synthetic Microbiology (SYNMIKRO), University of Marburg, Marburg, Germany

^7^ Institute of Pharmacology, University of Marburg, Marburg, Germany

^8^ Institute of Pharmaceutical Chemistry, University of Marburg, Marburg, Germany

*Address correspondence: [kornelia.hardes@ime.fraunhofer.de](mailto:kornelia.hardes@ime.fraunhofer.de)

**SUPPLEMENTARY MATERIAL**

**Table S1.** Furin homolog nucleotide sequences in NCBI.

| Gene | Organism | NCBI accession number | Nucleotide sequence length |
| --- | --- | --- | --- |
| Fur1 | *Aedes aegypti* | L46373.1 | 3,738 |
| Dfur1 | *Drosophila melanogaster* | L12376.1 | 3,801 |
| Dfur2 | *Drosophila melanogaster* | M94375.1 | 5,773 |
| FLP1 | *Aedes albopictus* | XM_062857281.1 | 4,245 |
| FLP2X1 | *Aedes albopictus* | XM_062857325.1 | 6,530 |
| FLP2X2 | *Aedes albopictus* | XM_062857326.1 | 6,520 |

**Table S2.** Homology identity (%) between furin gene sequences.

|  | Fur1 | Dfur1 | Dfur2 | FLP1 | FLP2X1 | FLP2X2 |
| --- | --- | --- | --- | --- | --- | --- |
| Fur1 |  | 55.618 | 0 | 63.675 | 46.154 | 48 |
| Dfur1 | 55.618 |  | 0 | 58.929 | 0 | 0 |
| Dfur2 | 0 | 0 |  | 36.496 | 58.253 | 58.383 |
| FLP1 | 63.675 | 58.929 | 36.496 |  | 41.958 | 42.097 |
| FLP2X1 | 46.154 | 0 | 58.253 | 41.958 |  | 99.557 |
| FLP2X2 | 48 | 0 | 58.383 | 42.097 | 99.557 |  |

**Table S3.** SFV6-2SG-mCherry passage information.

| Passage | Titer (PFU/mL) | Harvest time (h post-infection) |
| --- | --- | --- |
| 1 | 1.12E+07 | 48 |
| 2.A  2.B | 5.20E+07 2.42E+09 | 48  72 |

**Fig. S1**

**A**

**B**

**Fig. S1.** Inhibition of furin by inhibitors MI-1189 (A) and MI-1190 (B). Compound MI-1189 was characterized under tight-binding conditions in the presence of a single concentration (12.5 µM per well) of the substrate Phac-Arg-Val-Arg-Arg-AMC (0.95 nM furin per well, the data were fitted to equation 1, see main manuscript). Compound MI-1190 was characterized under classical conditions in the presence of the the same substrate at concentrations of 50 (●), 20 (●), and 5 (●) µM (0.95 nM furin per well, the data were fitted to equation 3, see main manuscript).

**Fig. S2**

**Fig. S2.** Cell viability of C6/36 and U4.4 cells in presence of the FITC-tagged inhibitor MI-1190. Confluent cells were treated with inhibitor MI-1190 (stock solution: 5 mM in 1:1 (v/v) H_2_O/DMSO, 100 µM per well). At 48 h post-treatment the cell viability was assessed via ATP quantification by CellTiter-Glo assay. The cell viability was normalized to the water control and expressed as a percentage (%). The mean cell viability (n=5) is shown and the error bards represent standard deviation (%). The dotted line represents the toxicity cut-off set at 80%.

**Fig. S3**

**Fig S3.** Toxicity of inhibitor MI-1851 applied with sheep blood erythrocytes (■) in Ae. albopictus female mosquitoes. The treatments (n=30) were individually provided to the female mosquitoes using the Hemotek Feeding System. Permethrin applied with erythrocytes (▲) and erythrocytes alone (🞐) were used as controls.

**Fig. S4**


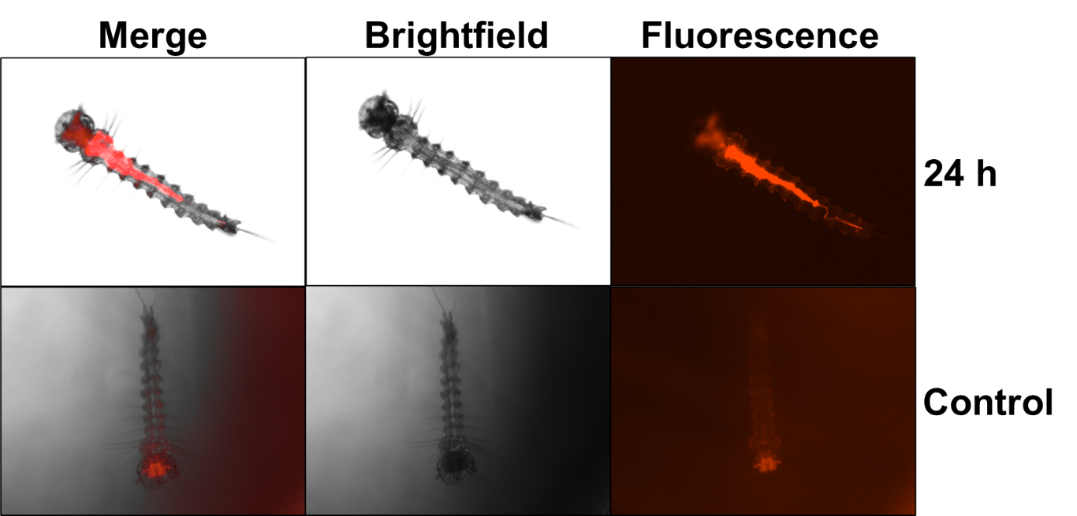


**Fig. S4.** Fluorescence analysis of reporter virus infection in *Ae. albopictus* L2 larvae. C6/36 cells were grown to confluence and infected with Semliki Forest virus-mCherry (SFV) at MOI of 0.01. At 48 h post-infection, larvae were placed in wells containing infected cells to feed on them. Fluorescence microscopy was performed to observe a confirming signal of virus infection emitted by SFV-mCherry at 24 h after infected cell digestion.

**Fig. S5**

**A**


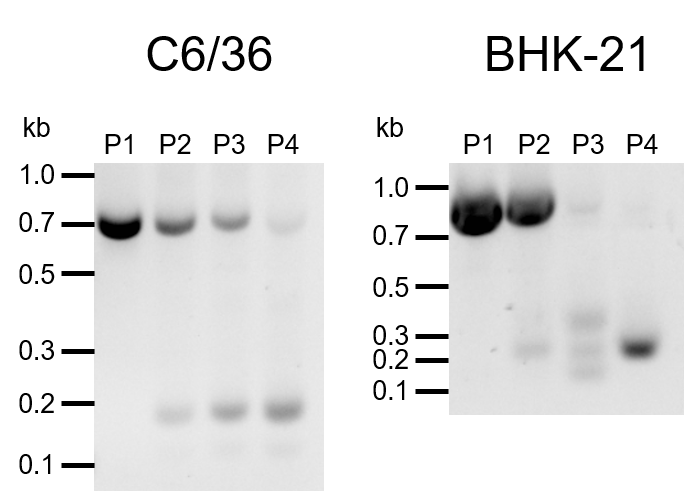


**B**


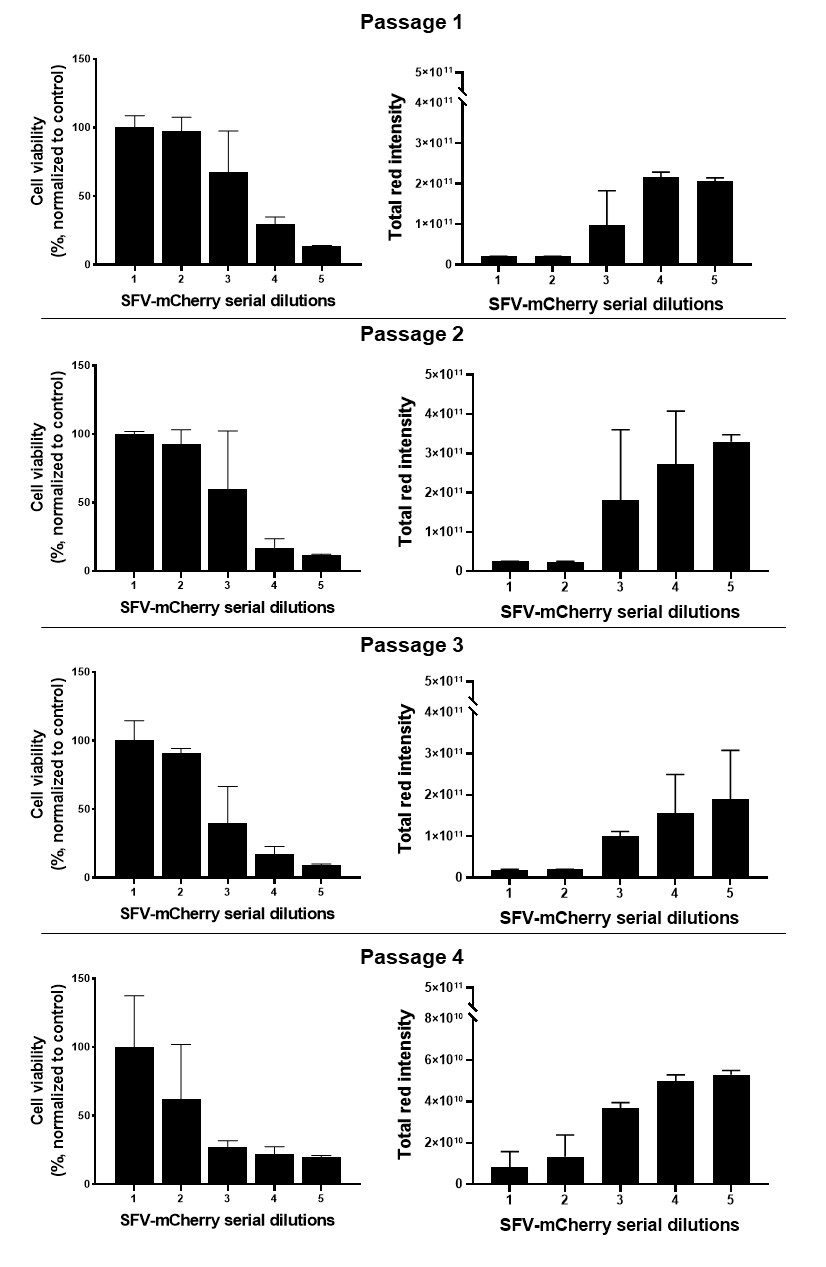


**Fig S5.** Reporter virus stability in C6/36 and BHK-21 cells. (A) RT-PCR fingerprint (primer sequences used: forward: ATGGTGAGCAAGGGCGAGGA, reverse: CTTGTACAGCTCGTCCATGC) of viral RNA extracted from virus-containing supernatants of SFV-mCherry infected C6/36 cells (left) and BHK-21 cells (right) over four passages. (B) Cell viability and total red intensity over four consecutive passages of the mCherry tagged Semliki Forest virus (SFV). BHK-21 cells were infected with SFV-mCherry (MOI: 0.001) for 1 h. At 48 h post-infection, the serial dilution of the SFV-mCherry passages was measured by their total red intensity and followed by cell viability quantification by CellTiter-Glo assay. The cell viability was normalized to the untreated control and expressed as a percentage (%). The mean total red intensity and cell viability (n=4) are shown and the error bars represent standard deviations (%).

**Fig. S6**

**Fig. S6.** Box and Whisker plot of the Ct-values of the selected housekeeping gene phosphatase-2A (PP2A) in the developmental stages of *Aedes albopictus* (L1-L4 instar larvae, pupae, sugar-fed female and male mosquitoes, as well as blood-fed (BF) females). Shown are the performances in the individual developmental stages as well as the overall performance. The ends of the box represent upper and lower quartiles; the median is marked by the horizontal line inside the box.

**Fig. S7**

**Fig S7.** Antiviral efficacy of the furin inhibitors upon SFV4 infection in C6/36 at 48 and 72 h post-infection. Cells were infected with SFV4 (MOI: 0.01) for 1 h. Furin inhibitors MI-1148, MI-1554, and MI-1851 were added after infection at concentrations of 0.1, 1, 10, and 100 µM. The data are mean values (n = 5 (48 h)/4 (72 h)) of the virus titer. Titer determination was performed by TCID_50_ assay on BHK-21 cells using standard methodology. Briefly, serial 10-fold dilutions of each sample were inoculated with the cells, which were incubated for 1 h under standard culture conditions. Following infection, cells were incubated for 48 hours at 37 °C, and the cytopathic effect was assessed using the CellTiter-Glo assay in accordance with the manufacturer’s instructions.

**Fig. S8**


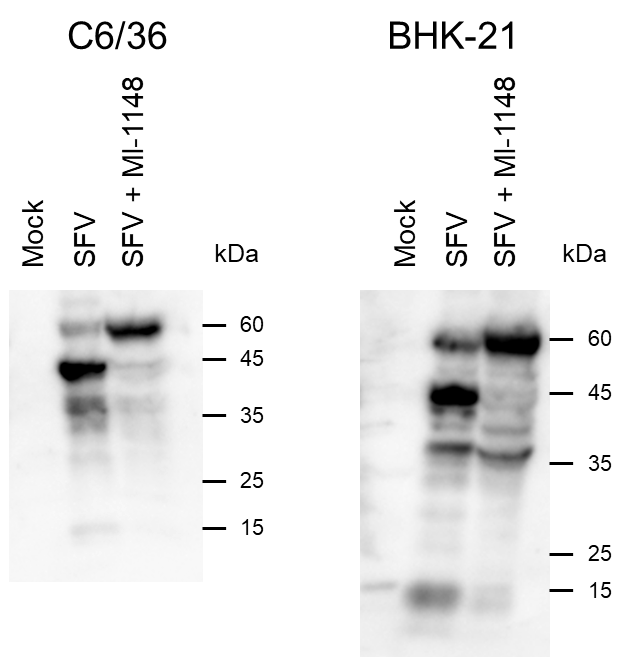


**Fig. S8: Inhibition of E3-E2 cleavage by inhibitor MI-1148 (25 μM in assay) compared to a control in the absence of inhibitor.** C6/36 cells and BHK-21 cells were infected with SFV4 at an MOI of 1 and incubated for 24 h. Proteins E3-E2 and mature E2 were immunochemically detected with an E2 antiserum after gel electrophoresis followed by Western blot analysis. Relative molecular mass of marker proteins displayed on the right-hand side.
